# Multi‐Functional Liposome: A Powerful Theranostic Nano‐Platform Enhancing Photodynamic Therapy

**DOI:** 10.1002/advs.202100876

**Published:** 2021-06-03

**Authors:** Xiamin Cheng, Jing Gao, Yang Ding, Yao Lu, Qiancheng Wei, Dezhi Cui, Jiali Fan, Xiaoman Li, Ershu Zhu, Yongna Lu, Qiong Wu, Lin Li, Wei Huang

**Affiliations:** ^1^ Institute of Advanced Synthesis School of Chemistry and Molecular Engineering Nanjing Tech University (NanjingTech) Nanjing 211816 P. R. China; ^2^ Key Laboratory of Flexible Electronics (KLOFE) & Institute of Advanced Materials (IAM) Nanjing Tech University (NanjingTech) Nanjing 211816 P. R. China

**Keywords:** combined therapy, drug delivery, liposomes, photodynamic therapy, theranostics

## Abstract

Although photodynamic therapy (PDT) has promising advantages in almost non‐invasion, low drug resistance, and low dark toxicity, it still suffers from limitations in the lipophilic nature of most photosensitizers (PSs), short half‐life of PS in plasma, poor tissue penetration, and low tumor specificity. To overcome these limitations and enhance PDT, liposomes, as excellent multi‐functional nano‐carriers for drug delivery, have been extensively studied in multi‐functional theranostics, including liposomal PS, targeted drug delivery, controllable drug release, image‐guided therapy, and combined therapy. This review provides researchers with a useful reference in liposome‐based drug delivery.

## Graphical abstract

Liposomes with high capacity for photosensitizers (PSs), other drugs and imaging agents in hydrophobic bilayer and hydrophilic aqueous core make them capable of executing multi‐functions in disease diagnosis and therapy to enhance photodynamic therapy. The flexibility of surface modification and components of liposome possess the advantages in targeted drug delivery and release.

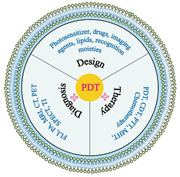

## 1 Introduction

**Figure 1:**
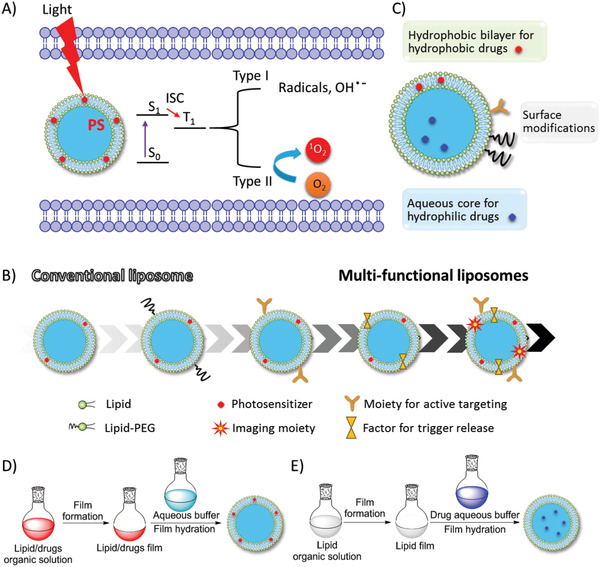
A) In the cell, liposomal PS is activated from the ground state (S_0_) to the excited state (S_1_) by light irradiation. Then, the singlet PS* is transferred to the triplet state PS* by ISC, the latter induced radicals and ROS (type I) and singlet oxygen (^1^O_2_) from triplet oxygen (^3^O_2_) (type II), which may cause cell damage. B) Development of multi‐functional liposomes. C) Typical liposome structure consist of the lipid bilayer, aqueous core, hydrophobic drugs imbedded in lipid bilayer, hydrophilic drug inside the aqueous core, recognition moieties and PEG linker on the liposome surface. D,E) Typical preparation encapsulation methods of PS and drugs: D) hydrophobic and E) hydrophilic.

Since porfimer sodium was approved as the first photosensitizer (PS) for the treatment of bladder cancer in 1993, photodynamic therapy (PDT) has been widely used in anti‐tumor and anti‐infection therapies.^[^ ^1^ ^]^ It is almost non‐invasive and non‐drug resistant than traditional cancer treatments (surgery, chemotherapy, and radiotherapy), owing to its particular mechanism of cytotoxicity to targets by the photochemistry induced by light‐activated PS. Upon absorption of light, PSs are excited to the excited singlet state (S_1_), and transform‐ to the triplet state (T_1_) by intersystem crossing (ISC). In the type I process, the excited PS at the T_1_ state transfers an electron to cellular substrates to form radicals and reactive oxidative species (ROS). In the type II process, there are three essential elements: PS, light, and oxygen. Singlet oxygen (^1^O_2_) is formed by transferring energy from photoexcited PSs at the T_1_ state to triplet oxygen (^3^O_2_), as shown in **Figure** **1**A. Although low levels of ROS are essential to regulate normal cell functions,^[^ ^2^ ^]^ high levels of ROS can disrupt the balance between ROS generation and detoxification, thus, interfering with biological systems, and causing cell damage by oxidizing biomolecules, such as lipids, amino acids, and heterocyclic bases in nucleic acids.^[^ ^3^ ^]^ The overall therapeutic effects of PDT include direct‐targeted cell ablation, vascular damage, and inflammatory and immune responses.^[^ ^4^ ^]^ Furthermore, ROS can only travel ≈10−55 nm upon excitation due to its short half‐life (≈10−320 ns) within cells.^[^ ^5^ ^]^ Therefore, PDT can be confined by the light‐focusing site and the distribution of PSs.

The delivery and bio‐distribution of singular PSs usually rely on their intrinsic characteristics and interactions with the physiological environment, which may greatly influence therapeutic effects. However, there remain major challenges in low efficiencies of targeted delivery, tissue penetration, and tissue accumulation of many PSs to target tissue/cell PDT. In vascular delivery, the biomolecules in the plasma and the body self‐clearance mechanism could obviously reduce the concentration of PS.^[^ ^6^ ^]^ PDT ablates the vessel to block the vesicular delivery of PSs to the tumor interior. The tissue penetration ability of light to activate PDT greatly limits the therapeutic depth. The lipophilic/hydrophobic nature of most PSs induces low solubility and aggregation in aqueous media, which not only decreases cellular uptake, but also increases the photothermal effect and quenches the generation of ROS.^[^ ^7^ ^]^ Although many PSs are fluorescent dyes and fluorescent imaging (FLI) allows the detection of the delivery, distribution, and metabolites of PS in cells, surface, and shallow tissue, the poor tissue penetration of optical imaging greatly limits its application in deep tissues and organs. In addition, the tumor microenvironment (TME) contributes to the tumor resistance to therapies, including PDT.^[^ ^8^ ^]^ For example, oxygen‐dependent PDT suffers from hypoxia, as the median oxygen levels of most tumors are below 2%.^[^ ^9^ ^]^ Additionally, oxygen consumption by PDT aggravates hypoxia. Hypoxia‐activated transcription factors, such as the transcription factor activator protein 1 (AP‐1), nuclear factor E2‐related factor 2 (NRF2), and hypoxia inducible factor 1 (HIF‐1), decrease the therapeutic effects of PDT, and may even promote the tumor invasion and metastasis.^[^ ^10^ ^]^ Some tumor cells increase their tolerance to high oxidative stress by upregulating the expression of glutathione (GSH) to neutralize ROS.^[^ ^11^ ^]^ Therefore, an efficient strategy to overcome the limitations of singular PSs is urgently needed for tumor therapy.

To date, many efforts have been made to overcome these limitations, such as supramolecular PS,^[^ ^12^ ^]^ activatable PS,^[^ ^13^ ^]^ nanoparticle delivery,^[^ ^14^ ^]^ and combined therapy.^[^ ^15^ ^]^ Among of these, liposomes have been widely applied in medicinal formulations in clinic and clinical trials for imaging, diagnosis, and therapy.^[^ ^16^ ^]^ Importantly, liposomes have the potential to overcome the limitations of singular PSs due to the following reasons: 1) Lipids are amphiphilic and can disperse PS to avoid aggregation and self‐quenching; 2) Liposomes are multi‐functional nano‐carriers for targeted delivery and triggered release on demand by multiple stimuli owing to the flexibility of modifications on their surface, size control, and tunable encapsulation;^[^ ^17^ ^]^ 3) Multi‐functional theranostics is possible by co‐loading other therapeutic agents and imaging contrasts. In addition, liposomes avoid the exposure of cargoes to normal tissue/organ and blood circulation, thus preventing the degradation of cargoes, immuno‐response, and possible toxicity, before reaching the desired lesion sites; 4) Liposomes consist of natural phospholipids, which are biocompatible and biodegradable materials, minimizes the risks induced by the formulation are minimized; and 5) Compared with free PSs, liposomal formulation of PSs improves therapeutic efficiency by prolonging tissue permeation and retention.

Currently, there are four excellent reviews on the early stages of liposomal formulations for PDT: Witte et al.^[^ ^18^ ^]^ in 2004, Zheng et al.^[^ ^19^ ^]^ in 2011, Azevedo et al.^[^ ^20^ ^]^ in 2011, and then Hamblin et al.^[^ ^21^ ^]^ in 2013. Since the liposomes for PDT have experienced several stages from conventional/simple to multi‐functional liposomes, we summarize the recent progress, especially the design and applications of the multi‐functional liposomes in theranostics (Figure 1B).

## 2 Construction of Liposomal Systems for PDT

### 2.1 Structure and Preparation Methods

As excellent drug carriers, liposomes are artificial lipid vesicles consisting of bilayer membranes and inner aqueous cores for the encapsulation of lipophilic/hydrophobic molecules/drugs and hydrophilic molecules/drugs, respectively. To overcome biological barriers that prevent the delivery of drugs to the targeted site,^[^ ^22^ ^]^ many efforts have been made in the construction of functional liposomes to enhance PDT efficacy, including self‐assembly of liposomes, surface modification, functional lipid assembly, and cargo loading (Figure 1C). The liposomal bilayer is mainly composed of natural or synthetic phospholipids, such as DMPC, DSPC and etc. as shown in **Table** **1**. Additionally, cell membrane capsules derived from cracked cancer cell membranes,^[^ ^23^ ^]^ red blood cell membranes,^[^ ^24^ ^]^ red blood cells,^[^ ^25^ ^]^ and extracellular vesicles,^[^ ^26^ ^]^ have been used in PDT. These materials retain some cell membrane channels and cytoplasmic functions and evade macrophage phagocytosis.^[^ ^27^ ^]^

**Table 1:** Characteristics of representative liposomal PSs

PS	*λ*_Ab_/*λ*_Em_ [nm]^c)^, ^d)^	*Ф*_FL_/*Ф*_△_^d)^, ^e)^	Diameters±SD [nm]	Phototoxicity	Components of liposome^c)^	Ref.
**PpIX**	409, 512, 548, 584, 637 (Water)	–/0.56 (Water)^[^ ^33^ ^]^	124± 0.85	IC_50_: 0.53 ± 0.19 × 10^−6^ m to Hela cells	PC	^[^ ^56^ ^]^
**PpIX‐DME**	404, 509, 539, 578, 630/627, 696(DMF)^[^ ^34^ ^]^	0.03 ± 0.05/0.67 ± 0.05(DMF)^[^ ^35^ ^]^	83.3 ± 6.7	IC_50_: 1.95 ± 0.31, 2.09 ± 0.81, 0.47 ± 0.28 × 10^−9^ m to C26, B16 and LLC tumor cells, respectively	PEG‐PLA	^[^ ^57^ ^]^
**TPPS_4_**	420, 552, 640/660 (PBS)	–/0.19(DMSO)^[^ ^36^ ^]^	111.0 ± 0.6	≈46% and ≈57% Hela cells survival	DPPC/DOTAP/DSPE‐PEG2000/cholesterol	^[^ ^58^ ^]^
**Ce6**	403, 664/652 (Water)^[^ ^37^ ^]^	0.18 (Water)^[^ ^37^ ^]^/ 0.63 ± 0.06 (DMF)^[^ ^38^ ^]^	114 ± 36	Toxicity to *C. albicans* ^b)^	DMPC/CTAB	^[^ ^59^ ^]^
**P18**	700/712(Ethanol)^[^ ^39^ ^]^	0.095/0.45 ± 0.10 (Ethanol)^[^ ^39^ ^]^	≈80	Toxicity to A549‐T tumor^b)^	DSPE‐PEG/DSPE‐PEG‐RGD/PC	^[^ ^60^ ^]^
**BPD**	^b)^/^b)^^[^^40^^]^	0.03/0.76 (Methanol)^[^ ^41^ ^]^	524.8^a)^	Toxicity to OVCAR5‐mCherry cells^b)^	PG/DMPC/ascorbyl palmitate/butylated hydroxytoluene	^[^ ^61^ ^]^
**mTHPC**	416, 650/652 (Methanol)^[^ ^42^ ^]^	0.089/(Methanol)^[^ ^42^ ^]^/ 0.68 ± 0.11 (DMF)^[^ ^38^ ^]^	135	Toxicity to CAL‐33 tumor^b)^	Lecithin/PEG surfactant	^[^^31^, ^62^^]^
**TATCPA**	590/670^[^ ^43^ ^]^	0.11 (Water)^[^ ^43^ ^]^/ –	137 ± 33^a)^	IC_50_: 1.17 × 10^−6^ m to A431 cells	EPC/cholesterol/PG	^[^ ^63^ ^]^
**Pc1**	741/750 (DMF), 670/760 (DMSO)	0.02/0.04 (DMF), 0.02/0.03 (DMSO)	381 ± 12	log reduction value 3.61 to *E. faecalis*	POPC/DOTAP,	^[^ ^65^ ^]^
**Pc2**	^b)^/750(DMF), ^b)^/760(DMSO)	0.10/0.14 (DMF), 0.03/0.13 (DMSO)	284 ± 9	log reduction value 2.99 to *E. faecalis*	POPC/DOTAP,	^[^ ^65^ ^]^
**ZnPc‐1**	741(DMF), 670(DMSO)	0.005/0.18(DMF), 0.004/0.16(DMSO)	174 ± 4	log reduction value 5.7 to *E. faecalis*	POPC/DOTAP	^[^ ^64^ ^]^
				log reduction value 0.5 to *E. Coli*		
**ZnPc**	674/678(Pyridine)	0.18/0.67 (DMSO)^[^ ^44^ ^]^	127 ± 17	Toxicity to Sk‐Cha1 Cells^b)^	DPPC/cholesterol/DSPE‐PEG	^[^^66^, ^67^^]^
**PCN**	673(DMF)/^b)^	–/^b)^	112	IC_50_: 0.049 ± 0.003 × 10^−6^ m to HeLa cells; 0.19 ± 0.02 × 10^−6^ m to MCF‐7 cells	DPPC/FA‐DSPE‐PEG_1k_/cholesterol	^[^ ^68^ ^]^
**Pz 1**	^b)^	–/0.05 (DMF)	280 ± 0.12	IC_50_: 0.26 × 10^−6^ m to CAL 27 cells; 0.19 × 10^−6^ m to HSC‐3 cells; 0.29 × 10^−6^ m to HeLa cells	DOTAP/POPC	^[^ ^69^ ^]^
**Pz 2**	^b)^	–/0.20(DMF)	360 ± 0.19	IC_50_: 0.007 × 10^−6^ m to CAL 27 cells; 0.013 × 10^−6^ m to HSC‐3 cells; 0.042 × 10^−6^ m to HeLa cells	DOTAP/POPC	^[^ ^69^ ^]^
**Pz 3**	^b)^	–/0.15(DMF)	220 ± 0.08	IC_50_: 0.08 × 10^−6^ m to CAL 27 cells; 1.82 × 10^−6^ m to HSC‐3; 5.06 × 10^−6^ m to HeLa cells	DOTAP/POPC	^[^ ^69^ ^]^
**Pz1**	658, 689/707(DMF), 658, 692/708(DMSO)	0.026/0.281 ± 0.006 (DMF), 0.045/0.311 ± 0.003 (DMSO)	–	IC_50_: 129 × 10^−9^ m to HSC‐3 cells	Free	^[^ ^71^ ^]^
			123.4 ± 32	IC_50_: 45 × 10^−9^ m to HSC‐3 cells	PG/POPC	
**Porphyrazine 5**	352, 657, 695 (DCM) /683, 699 (DMF); 683, 792 (DMSO)	0.19/0.069 ± 0.001 (DMF), 0.16/0.180 ± 0.006 (DMSO)	80 ± 170/400 ± 290	IC_50_: 0.600 ± 0.357 × 10^−6^ m to LNCaP cells	DOTAP/POPC	^[^ ^72^ ^]^
			70 ± 130, 400 ± 280	IC_50_: 0.378 ± 0.002 × 10^−6^ m to LNCaP cells	PG/POPC	
**ClAlPc**	^b)^/630, 750	–/0.29 (DMSO), 0.69 (Liposome)	–	80% metastatic melanoma cells death at 700 mJ cm^−2^	–	^[^^73^, ^74^^]^
**AlPcS4**	^b)^/^b)^	–/^b)^	158.1	20% cell viability of SGC‐7901 cells at 8 µg mL^−1^.	DPPC/DOTAP/cholesterol/PEG2000‐DSPE	^[^ ^75^ ^]^
**Hyp**	544/587 (Methanol)	–/0.39±0.01 (Methanol)^[^ ^45^ ^]^	127 ± 14	IC_50_:48 × 10^−9^ m to SK‐OV‐3 cells	DPPC/TEL	^[^ ^76^ ^]^
			127 ± 12	1.9 – 2 log reduction after 30 min and 2.3 – 2.5 log reduction after 120 min to *S. saprophyticus subsp. bovis*	DOPE/DPPC/CHEMS	^[^ ^77^ ^]^
			126 ± 19		DSPC/DPPC/DSPE‐PEG	
			173 ± 21		DPPC/DOTAP	
**Hypocrellin B**	658/‐(DMF)^[^ ^46^ ^]^	–/0.74^[^ ^46^ ^]^	93 ± 16	IC_50_: 101 × 10^−9^ m to RMVECs	egg lecithin/cholesterol	^[^^79^, ^80^^]^
**PBDP‐VER**	675/730	–/0.46(DMSO)	105.4	IC_50_: 0.88 × 10^−6^ m to SK–OV‐3 cells	EPC/DOPE	^[^ ^82^ ^]^
**NBDP‐VER**	684/725	–/0.37(DMSO)	111.3	IC_50_: 0.35 × 10^−6^ m to SK‐OV‐3 cells	EPC/DOPE	^[^ ^82^ ^]^
**NBDP‐SAL**	664/695	–/0.40(DMSO)	106.7	IC_50_: 0.65 × 10^−6^ m to SK‐OV‐3 cells	EPC/DOPE	^[^ ^82^ ^]^
**NBDP‐NVER**	657/700	–/0.80(DMSO)	102.9	IC_50_: 0.86 × 10^−6^ m to SK‐OV‐3 cells	EPC/DOPE	^[^ ^82^ ^]^
**DiBDP**	511	–/0.05 to 0.46	86 ±17	Toxicity to HeLa cells^b)^	DOPE/CHEMS/DSPE‐mPEG2000	^[^ ^83^ ^]^
**C60‐2**	^b)^	–/^b)^	138^a)^	IC_50_:1.1 × 10^−6^ m to HeLa cells	DMPC/DTAB	^[^^84^, ^85^^]^
**R6G**	526/550 (Water), 530/554(Liposome)	–/0.308(Water)	–	–	Free	^[^ ^86^ ^]^
		–/0.614(PR6G)	172.1^a)^	Toxicity to *P. aeruginosa* ^b)^	Egg phospholipid/cholesterol/PVA	
		–/0.6022 (CR6G)	126.3^a)^	Toxicity to *P. aeruginosa* ^b)^	Egg phospholipid/cholesterol/CTAB	
**MB**	660/–	–/^b)^	140 ± 35	Toxicity to 4T1 cells^b)^	DPPE‐PCB/DSPC	^[^^87^, ^88^^]^
**ICG**	780/820	0.043 (Methanol)^[^ ^47^ ^]^ /0.01 (Ethanol)	71 ± 10	Toxicity to MDA‐MB‐468, HCC‐1806 cells^b)^	DPPC/SoyPC/Cholesterol/DSPE‐PEG 2000	^[^^90^, ^91^^]^
**BP**	–/^b)^	–/^a)^, ^b)^	160 – 200	Toxicity to MCF‐7 cells^b)^	DMPC/DSPE‐PEG2000/DSPE‐PEG‐Folate	^[^ ^94^ ^]^
**PpIX‐Ole**	405 (S), 507, 541, 577, 631 (Q) (Chloroform)	–	140	Toxicty to HeLa and AGS cells^b)^	DOPC	^[^ ^95^ ^]^
**PL‐C17**	637(Water)	–	149.9 ± 2.6/ 176.9 ± 1.3	IC_50_: 12.9 ± 1.13 × 10^−6^ m to HeLa cells	DSPC/PEG2000‐DSPE/cholesterol	^[^ ^96^ ^]^
**PhLPC**	410, 667 (20) (Solvents mixture)	–	168.1 ± 15.0	IC_50_: 2.95 ± 0.14 × 10^−6^ m to HET‐1A cells; 1.90 ± 0.06 × 10^−6^ m to Kyse‐30 cells	DSPC/DSPE‐PEG2000	^[^ ^97^ ^]^
**PhLSM**	406, 667 (21) (Solvents mixture)	–	191.5 ± 31.4	IC_50_: 2.04 ± 0.15 × 10^−6^ m to HET‐1A cells; 1.00 ± 0.04 × 10^−6^ m to Kyse‐30 cells	DSPC/DSPE‐PEG2000	^[^ ^97^ ^]^
**PC‐BPD**	–	–	106.1^a)^	Toxicity to OVCAR5‐mCherry cells^b)^	DPPC/DOTAP/DSPE‐PEG2000	^[^ ^61^ ^]^
**AIEgen‐lipid**	≈430/≈610	0.15 ± 0.01/0.88(Liposome)	100	IC_50_: 9.7 µg mL^−1^ to 4T1 cells	DSPE‐PEG2000/DSPC	^[^ ^93^ ^]^
**iDOPE**	786/811 (Methanol)	‐/0.0062(Chloroform)	191	–	DOPC	^[^ ^89^ ^]^
**ICG‐C18**	^b)^ ^[^ ^48^ ^]^	–	235 ± 101	Toxicity to SCCVII tumor cells^b)^	DOPE/cholesterol/phosphatidylethanolamine‐N‐methoxy‐polyethylene glycol(5000)‐dioleoyl‐glycero ammonium salt	^[^ ^98^ ^]^
**CuInS_2_/ZnS**	–	–/^b)^	37.43 ± 15.76	Toxicity to Eca‐109 cells^b)^	soybean phospholipids/DSPE‐PEG2000‐NH_2_	^[^ ^99^ ^]^

^a)^ Average hydrodynamic diameters D_hy_ (nm);

^b)^ The literature showed the figure without accurate data;

^c)^ 1‐Palmitoyl‐2‐oleoyl‐sn‐glycero‐3‐phospho‐choline (POPC); L‐*α*‐phosphatidyl‐D,L‐glycerol (chicken egg, PG); 1,2‐distearoyl‐sn‐glycero‐3‐phosphatidylcholine (DSPC); phospholipid L‐*α*‐1,2‐dipalmitoyl‐sn‐glycero‐3‐phosphocholine (DPPC); egg L‐*α*‐phosphatidylcholine (EPC); 1,2‐dioleoyl‐sn‐glycero‐3‐phosphoethanolamine (DOPE); cholesteryl hemisuccinate (CHEMS); 1,2‐distearoyl‐sn‐glycero‐3‐phosphoethanolamine‐N‐[methoxy(polyethyleneglycol)‐2000 (DSPE‐mPEG2000); 1,2‐dimyristoyl‐sn‐glycero‐3‐phosphocholine (DMPC); N,N‐dihexadecyl‐N^*α*^‐[6‐(trimethylammonio)hexanoyl]‐L‐aspartamide bromide (DTAB); 1,2‐dipalmitoyl‐sn‐glycero‐3‐phosphoethanolamine (DPPE‐PCB); polyvinyl alcohol (PVA); cetrimonium bromide (CTAB); 1,4‐diphenylisobenzofurane (DPBF); poly(ethylene glycol)‐block‐polylactic acid (PEG‐PLA). 1,2‐dioleoyl‐3‐trimethylammonium‐propane (DOTAP), distearoyl phosphoethanolamine modified with folic acid (FA‐DSPE‐PEG_1k_) and egg phosphatidylcholine (PC);

^d)^ Maximum absorption peak, maximum emission peak, and reference are abbreviated as ab, em, and ref, respectively;

^e)^ Quantum yield of fluorescence: *Ф*_FL_; quantum yield of ROS: *Ф*_△_.

Various methods have been developed to prepare liposomes encapsulating payloads for multiple purposes, such as thin‐film hydration, reverse‐phase evaporation, and solvent injection.^[^ ^16a^ ^]^ The most widely used method is lipid film hydration. Hydrophobic PS, drugs and lipids are dissolved in organic solvents and dried to form thin film, which is then hydrated by aqueous media (Figure 1D); on the other hand, hydrophilic PS and drugs dissolved in aqueous media are encapsulated by lipid film to form the aqueous core (Figure 1E). The resulting unilamellar vesicles (ULVs) have heterogeneous sizes and are often accompanied by multilamellar vesicles (MLVs). To control the size and homogeneity of final liposomes, a subsequent process is required, such as membrane extrusion, sonication, or high‐pressure homogenization. As one of the most common methods, the membrane extrusion method reduces and homogenizes the size of large and heterogeneous ULVs and MLVs by repeatedly pushing them through a membrane filter with a homogeneous pore size. Prior to extrusion, the formed large MLVs are disrupted by several cycles of freeze‐thaw or prefiltration. The extrusion process should be operated at a temperature above the phase transition temperature of the lipid to reduce rigidness. In addition, large MLVs could be disrupted by sonication to produce small ULVs, which are then purified by centrifugation. In high‐pressure homogenization, large ULVs and MLVs are reduced to small ULVs through turbulence and hydraulic shear under high pressure after repeated recirculation.

The components and size of liposomes influence their stability and drug delivery. Cholesterol is often used as an additive in liposomes to induce the stability and drug release.^[^ ^28^ ^]^ Hydrogen bonding between the head of the lipid and PS affects the rigidity of liposomes, which is responsible for their capacity.^[^ ^29^ ^]^ Not only free molecular cargoes, but also sub‐containers with cargoes, such as MSN,^[^ ^30^ ^]^ macromolecule (cyclodextrin),^[^ ^31^ ^]^ nanoparticles (layered double hydroxide, LDH),^[^ ^32^ ^]^ can be encapsulated into liposomes.

### 2.2 Liposomal PSs

The structure and intrinsic characteristics of PS are key factors for PDT. Therefore, the efforts toward ideal PS mainly focused on improving the quantum yield of ROS and optimizing the absorption of PS into the therapeutic window (near infrared (NIR) region: 600–800 nm or longer wavelength) for better tissue penetration and high absorption efficiency to reduce the dose of PS.^[^ ^49^ ^]^ The liposomal formulation of PSs helps to successfully overcome the limitations of the singular PS in PDT (Table 1).

#### 2.2.1 Typical Liposomal PSs

As the most successful unit of PSs, many porphyrinoids have been investigated and have entered clinical and preclinical trials including the 1^st^, 2^nd^, and 3^rd^ generations of PSs.^[^ ^50^ ^]^ Because most porphyrinoids are hydrophobic, liposomal formulations improve water solubility and cell uptake. Heger et al. reviewed liposomal 2^nd^ generation PS for the treatment of solid cancers.^[^ ^51^ ^]^ Skupin‐mrugalska et al. reviewed the status of liposomal porphyrinoids as PSs and PS precursor (5‐aminolevulinic acid, **5‐ALA**).^[^ ^52^ ^]^ Recently, Duzgunes et al. reviewed the works on phorphyrinoid derivatives.^[^ ^53^ ^]^ Here, we focus on the application of new developments.

**Figure 2:**
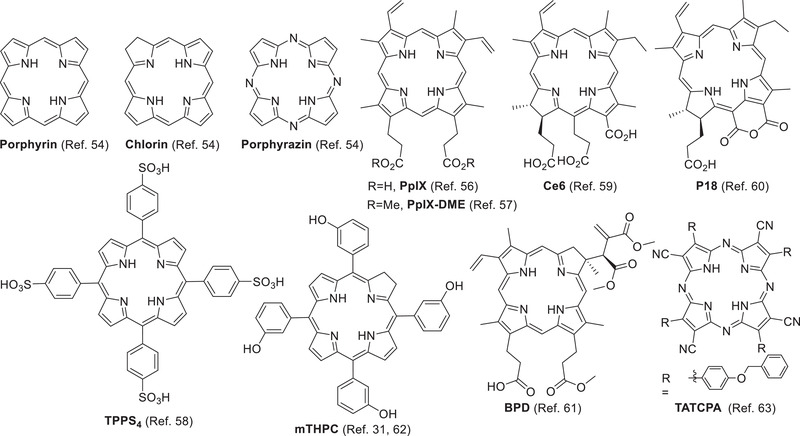
Representative parent porphyrinoids and liposomal porphyrinoids.

Porphyrinoids containing a conjugating macrocyclic tetrapyrrolic core has a high co‐efficiency in absorption of visible light and good photosensitivity.^[^ ^54^ ^]^ Most porphyrinoids are derivatives of three types of structures: **porphyrin**, **chlorin**, and **porphyrazin** (**Figure** **2**). For the phorphyrinoid system, the Q band attributed to the S_0_ to S_1_ transition is the key parameter. The extension of the conjugation system by the benzene ring and C═C bond normally results in a bathochromic shift, which means a longer wavelength of absorption for better tissue penetration. The Q band at 680 nm of **chlorin** is also redshifted from that at 580 nm of **porphyrin** as its parent structure. A reduced exocyclic double bond on **chlorin** decreases the symmetry of the conjugated macrocycle, leading to increased absorption in the long‐wavelength portion of the visible region.^[^ ^55^ ^]^

The porphyrin derivatives photoprotoporphyrin IX (**PpIX**), photoprotoporphyrin IX dimethyl ester (**PpIX‐DME**), and **TPPS_4_** with longer wavelengths were incorporated into liposomes for tumor therapy (Figure 2). Due to its hydrophobic nature, the aqueous **PpIX** forming crystals were internalized into cancer cells. However, the liposomal formulation of **PpIX** improved its internalization and bio‐distribution in HeLa cells.^[^ ^56^ ^]^ **PpIX‐DME** was also encapsulated in liposomes (PN‐Por) for tumor therapy.^[^ ^57^ ^]^ Liposomal **TPPS_4_** showed higher efficiency than free **TPPS_4_** with ≈46% and ≈57% HeLa cell survival rate under light irradiation at 552 and 640 nm, respectively.^[^ ^58^ ^]^

Chlorin derivatives chlorin e6 (**Ce6**),^[^ ^59^ ^]^ m‐tetrahydroxyphenylchlorin (**mTHPC**),^[^ ^31^ ^]^ purpurin 18 (**P18**),^[^ ^60^ ^]^ and benzoporphyrin derivative monoacid A (**BPD**)^[^ ^61^ ^]^ have been encapsulated in liposomes for tumor therapy (Figure 2). Chen et al. prepared cationic CTAB‐liposomes encapsulated with **Ce6** in various ratios of DMPC and cetyltrimethylammonium bromide (CTAB) as cationic surfactants.^[^ ^59^ ^]^ Different formulations of liposomal **mTHPC**, such as Foscan, Foslip, and Fospeg, have been successfully employed for various tumor treatments.^[^ ^62^ ^]^ Bezdetnaya et al. fabricated a novel hybrid delivery system called Temoporfin‐in‐Cyclodextrin‐in‐Liposome,^[^ ^31^ ^]^ in which **mTHPC** was bound to *β*‐CDs located in the inner aqueous core. **P18** nanoconfined liposomes (P18⊂L) were prepared to treat Taxol‐resistant tumor cells by suppressing cancer cell migration and destroying metastasis‐related extracellular matrix (ECM) in PDT.^[^ ^60^ ^]^

The liposomal tetra(aryl)‐tetracyanoporphyrazine (**TATCPA**) showed a high rate of cell uptake and strong phototoxicity in tumor PDT (Figure 2).^[^ ^63^ ^]^ The hydrophobic PS showed environment‐sensitive fluorescent enhancement and a hypsochromic shift in liposomes.

**Figure 3:**
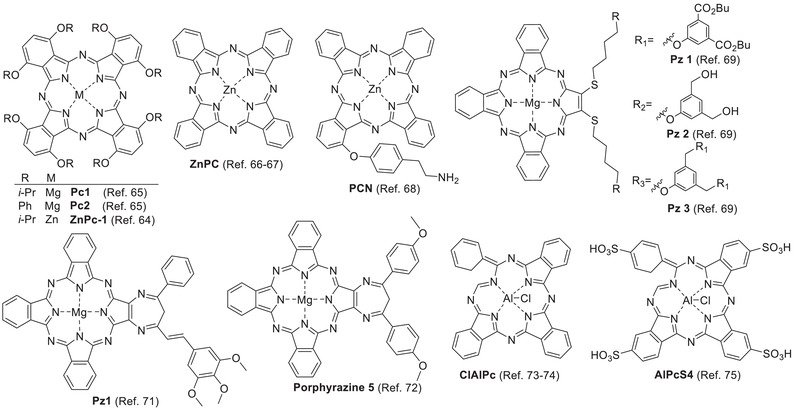
Liposomal porphyrinoids with metal chelation.

Chelation of metal ions to porphyrinoids, such as Zn^2+^, Mg^2+^, and Al^3+^, may affect their photophysical properties for better PDT performance (**Figure** **3**).^[^ ^55^ ^]^ Owing to the heavy atom effect preferring to ISC, the **ZnPc** derivative **ZnPc‐1** has a lower Ф_FL_ but higher Ф_△_ than Magnesium(II) Phthalocyanine **Pc1**. In the liposomal formulation, **Pc1** showed the highest inhibition against *E. faecalis* with 3.61 log reduction, in contrast to the zinc(II) analogue **ZnPc‐1** (5.70 log),^[^ ^64^ ^]^ which is consistent with their quantum efficiencies.^[^ ^65^ ^]^ **ZnPc** was also encapsulated in DPPC liposomes for HeLa^[^ ^66^ ^]^ and Sk‐Cha1 cells.^[^ ^67^ ^]^ In a model of protein oxidation, ROS produced by liposomal **ZnPc** can oxidize the autofluorescent amino acid Trp on BSA, which is detected quantitatively. An analog of **ZnPc**, **PcN**, was encapsulated into the nanoliposome PcN@Lip‐FA, which significantly improved cellular uptake and phototoxicity against HeLa cells.^[^ ^68^ ^]^

Liposomal dendritic magnesium sulfanyl tribenzoporphyrazine **Pz 1–3** as PSs^[^ ^69^ ^]^ activated by NIR irradiation demonstrated significantly better phototoxicity in cancer cell lines (Figure 3).^[^ ^70^ ^]^ Diazepinoporphyrazines modified with peripheral styryl groups were encapsulated in liposomes for PDT against oral cancer cell lines.^[^ ^71^ ^]^ The photocytoxicity of **Pz1** was increased by nearly 3‐fold (from IC_50_ 45 nm to IC_50_ 129 nm) compared with that of the solution of **Pz1**. Liposomal **porphyrazine 5** showed better photocytotoxic effects (DOTAP‐POPC‐5, IC_50_ = 0.600 ± 0.357 × 10^−6^ m; and PG‐POPC‐5, IC_50_ = 0.378 ± 0.002 × 10^−6^ m) than its free form (IC_50_ = 3.135 ± 0.156 × 10^−6^ m) to LNCaP cells under hypoxia and moderate phototoxicity at 5 × 10^−5^ × 10^−6^ m for antibacterial therapy.^[^ ^72^ ^]^

Liposomal chloroaluminum phthalocyanine (**ClAlPc**) has been used in PDT of non‐metastatic melanoma cells^[^ ^73^ ^]^ and human metastatic melanoma cells (Figure 3).^[^ ^74^ ^]^ Notably, its liposomal formulation improved not only the drug delivery to the desired site, but also the PS's photochemical properties of PS. The triplet state lifetime (*τ*_T,_ 2.63 µs) of liposomal **ClAlPc** was much longer than that (0.8 µs) of free **ClAlPc** in DMSO, resulting in a higher quantum yield (Ф_△_: 0.69 vs 0.29). **AlPcS4** has better water solubility and poor cell permeability than **ClAlPc** due to the four sulfonic acid groups. Cationic liposomes enhanced the delivery efficiency of **AlPcS4** and blocked its high affinity to serum albumin (Figure 3).^[^ ^75^ ^]^

**Figure 4:**
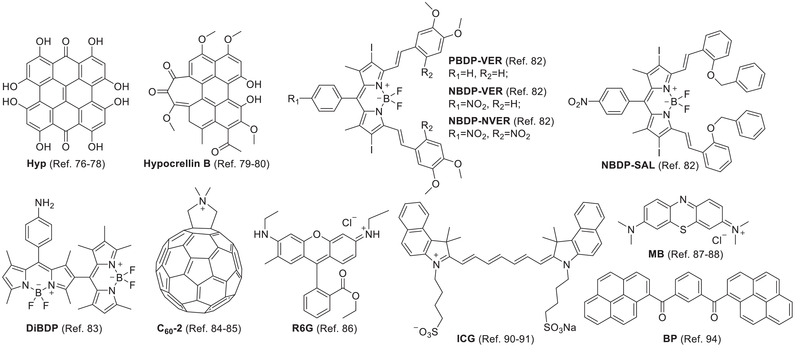
Other types of liposomal PSs.

Liposomal hypericin (**Hyp**) exerts antitumor, antiangiogenic effects^[^ ^76^ ^]^ and *anti*‐Gram‐positive bacteria activity.^[^ ^77^ ^]^ Caeano et al. studied Hyp in DPPC liposomes (**Figure** **4**).^[^ ^78^ ^]^ **Hypocrellin B**, isolated from the natural fungus sacs of *Hypocrella bambuase*, has high photosensitizing ability and low dark toxicity (Figure 4).^[^ ^46^ ^]^ However, its high metabolic rate and hydrophobicity hinder further clinical application.^[^ ^79^ ^]^ By improving in‐vivo stability and cell uptake, liposomal **hypocrellin B** showed potential as treatment for age‐related macular degeneration.^[^ ^80^ ^]^

BODIPY‐based PSs have been widely investigated for PDT.^[^ ^81^ ^]^ Compared with conventional BOIDPY dye, the halogen‐BODIPY dye has strong photosensitivity due to the spin‐orbit coupling by the heavy atom (e.g., Br and I), which greatly enhances the ISC. The flexible modification of BODIPY extends the absorption wavelength to the therapeutic window (650–800 nm) for in vivo applications. Although PSs have advantages such as good photo‐stability, high quantum yield, high molar extinction coefficient, and sharp absorption peak, hydrophobicity and lack of tumor‐specificity still limit their further application in PDT. By encapsulation in liposomes, Singh reported that a group of halogen‐BODIPY dyes (**NBDP‐VER**, **NBDP‐SAL**, etc.) for PDT has better toxicity against human ovarian carcinoma cell line (SK‐OV‐3) with an IC_50_ of 0.350 × 10^−6^ m (Figure 4).^[^ ^82^ ^]^ to halogened BODIPY, dimeric BODIPY (**DiBDP**) also showed good photosensitivity (Figure 4).^[^ ^83^ ^]^

A lipid‐membrane‐incorporated fullerene (C_60_) derivative LMIC_60_‐2 bearing a polar cation group showed much better photodynamic activity than other analogues with pristine C_60_ (Figure 4).^[^ ^84^ ^]^ LMIC_60_ prepared by the guest exchange method had much higher stability, water solubility, and photodynamic activity than that by the conventional method.^[^ ^85^ ^]^ To solve the problem of low sensitivity in the wavelength range from 600 to 700 nm, a light‐harvesting antenna molecule DiD was co‐loaded.

As a PS and fluorophore, Rhodamine 6G (**R6G**) was encapsulated in cationic liposomes for the treatment of multidrug‐resistant Gram‐negative bacteria in sewage treatment plants (Figure 4).^[^ ^86^ ^]^ The cationic liposome formulation **R6G** displayed better antibacterial efficiency than the neutral formulation PR6G due to the negative charge on the surface of the Gram‐negative bacteria.

Methylene blue (**MB**) based on a phenothiazinium scaffold has strong photosensitizing ability and NIR absorbance at 650–670 nm for better tissue penetration (Figure 4). However, its good hydrophilicity (water solubility) led to poor membrane penetration and cell uptake. The liposomal formulation improved cell uptake, reduced dark toxicity, and increased phototoxicity to 4T1 cells.^[^ ^87^ ^]^ The liposomal formulation improved the skin penetration of **MB** gel in the treatment of truncal acne vulgaris.^[^ ^88^ ^]^

Because of the long polymethine bridge, indocyanine green (**ICG**) has NIR absorbance and NIR fluorescence for optical imaging (Figure 4).^[^ ^89^ ^]^ In addition, its low dark toxicity and high phototoxicity suitable for generating heat and ROS upon NIR irradiation enable its applications in photothermal therapy (PTT) and PDT.^[^ ^90^ ^]^ To overcome the drawbacks of aggregation, rapid body clearance, and short residence time in blood, liposomal **ICG** was applied to PDT of human triple‐negative breast cancer cells.^[^ ^91^ ^]^ However, compared with free **ICG**, some liposomal **ICG**s displayed negligible ROS production, which was possibly attributed to ROS absorption by nearby phospholipids and **ICG** molecules.^[^ ^90b^ ^]^ More studies discussed the use of liposomally formulated **ICG** in optical imaging and cancer treatments including PTT and PDT in Sections 3.4 and 3.5.

**Figure 5:**
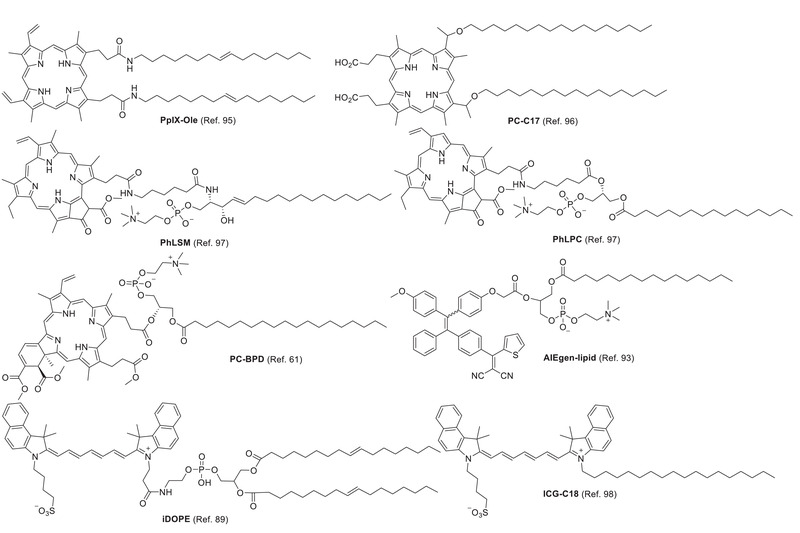
Lipid‐conjugated PSs.

Unlike aggregation‐caused quenching (ACQ) PS, AIEgen does not suffer from self‐quenching in fluorescence and photosensitivity limitations due to its special aggregation‐induced emission (AIE) enhancement phenomenon.^[^ ^92^ ^]^ We developed an “AIEsome” by self‐assembly of AIEgen‐conjugated lipid (**AIEgen‐lipid**) in liposomes for imaging‐guided PDT (**Figure** **5**).^[^ ^93^ ^]^ Subsequently, Li et al. reported that liposomal AIE‐PS (**BP**) could be activated by re‐aggregation of **BP** following liposome disruption in the tumor (Figure 4).^[^ ^94^ ^]^

To avoid PS leakage from liposomes, lipids are covalently conjugated to PS, thus improving affinity to the liposomal bilayer. Conjugation dose not significantly change the electron absorption and photosensitivity of PS. For instance, as building blocks, conjugates of lipid‐porphyrin (**PpIX‐Ole**,^[^ ^95^ ^]^ **PL‐C17**,^[^ ^96^ ^]^ **PhLPC**, **PhLSM**,^[^ ^97^ ^]^ and **PC‐BPD** ^[^ ^61^ ^]^), **AIEgen‐lipid**,^[^ ^93^ ^]^ and lipid‐ICG (**iDOPE** ^[^ ^89^ ^]^ and **ICG‐C18** ^[^ ^98^ ^]^) were incorporated into liposomes by self‐assembly (Figure 5). Free PS and lipid‐PS conjugates encapsulated in liposomes induce cell death via different pathways. For example, after their release from liposomes, the bio‐distribution of free **BPD** in the mitochondria and endoplasmic reticulum (ER) resulted in mitochondrial‐related cell death in PDT. However, liposomal **PC‐BPD** was endocytosed to form endosomes and then lysosomes, resulting in lysosomal photodamage in PDT.^[^ ^61^ ^]^

Inorganic materials have also been successfully used in liposome‐based PDT. Normally, inorganic PS displays high satiability, high sensitizing efficiency, high photothermal effect, and activation by NIR light. **CuInS_2_/ZnS** nanocrystals (quantum dots) were incorporated into the liposomes for PDT.^[^ ^99^ ^]^ However, the leakage of toxic heavy metal ions (Cu^2+^) hinders their biomedical application. Liposomal absorber reduced graphene oxide (rGO) significantly constrained the leakage. In another case, **d‐TiO_2_** NPs exhibited a broad absorption area from 400 to 1000 nm and photosensitivity upon irradiation with visible light (400–800 nm).^[^ ^100^ ^]^ **TiO_2_** has good stability and biocompatibility in physiological environments. The liposome‐TiO_2_ composites showed photo‐toxicity to cancer cells (HeLa and HepG2).

In general, the amphiphilic properties of liposomes make them suitable for the delivery of all kinds of PSs and have many promising advantages, regardless of the hydrophobic or hydrophilic nature of the drugs.

#### 2.2.2 Activatable Liposomal PSs

**Figure 6:**
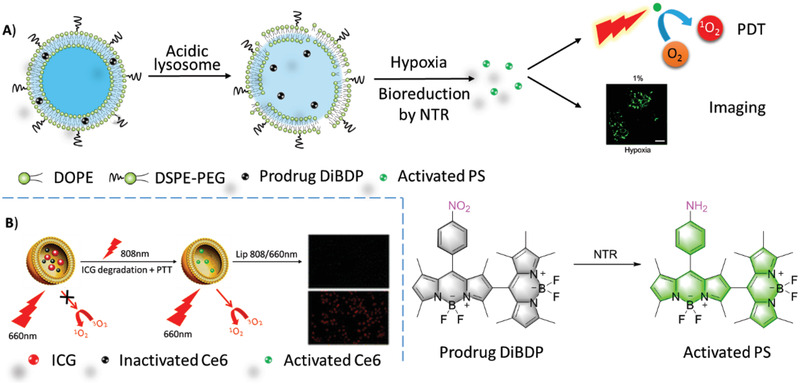
A) Upon the liposomes were collapsed in acidic lysosome, due to the degradation of acid‐sensitive lipid DOPE, liposomal **DiBDP** was activated by NTR under hypoxia. Confocal fluorescence microscopy imaging of HeLa cells which were pre‐cultured under hypoxic (1% *p*O_2_) conditions for 6 h and then incubated with Ab‐DiBDP NPs (50 mg mL^−1^) for 20 min. *λ*_ex_ = 543 nm, Scale bar = 40 µm. Reproduced with permission.^[^ ^83^ ^]^ Copyright 2018, Royal Society of Chemistry. B) Schematic illustration of combined therapy by liposomal system: Lip(**Ce6**+**ICG**). Reproduced with permission.^[^ ^101^ ^]^ Copyright 2015, Royal Society of Chemistry.

To reduce non‐specific damage to normal cells, PSs are silenced, even under light irradiation before they reach the targeted site and recover their photosensitivity on demand under external or internal stimuli.^[^ ^13^ ^]^ The prodrug **DiBDP** was released at the acidic lysosome and then activated by intracellular NTR in hypoxia only to turn on phototoxicity and fluorescence (**Figure** **6**A).^[^ ^83^ ^]^ Fluorescence resonance energy transfer (FRET) is a typical method for PS regulation. **ICG** ^[^ ^101^ ^]^ or DiR^[^ ^102^ ^]^ quenched the liposomal **Ce6** by FRET. The photoactivity of **Ce6** was recovered upon **ICG** or DiR was destroyed by NIR laser irradiation (Figure 6B).

### 2.3 Functional Liposomes

To deliver and release the cargo to the desired site, liposomes have to overcome many challenges, such as plasma stability, specificity, internalization, and drug release. These concerns have been addressed using the functional components immobilized on the surface or imbedded in the lipid layers.^[^ ^103^ ^]^ For example, PEG modification improves the stability of liposomes in aqueous media and the half‐life of circulation in vivo by reducing body clearance, non‐specific interactions with physiological biomolecules, and immuno‐attacks. However, crowded PEG chains on liposomes can hinder their approach to tumors.^[^ ^104^ ^]^ This is solved by cleavable PEG^[^ ^105^ ^]^ and Zwitterionic liposomes.^[^ ^87^ ^]^ More strategies are introduced as follows.

#### 2.3.1 Active‐Targeted Liposomes

**Figure 7:**
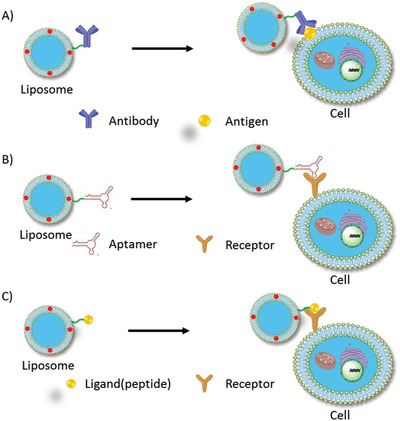
Typical models of active‐targeted liposomes. Specific recognitions includes: A) antibody‐antigen interactions, B) aptamer‐receptor interactions and C) ligand‐receptor interactions.

Because PDT is restricted by the bio‐distribution of PS as the short radius of ROS traveling, liposomal PS needs to be delivered and accumulated in target sites (e.g., tumor cells/tissues). In conventional liposomes, liposomal PSs are delivered passively to the tumor by enhanced permeability and retention (EPR) effect; however, their specificity is insufficient. In PDT, the unavoidable accumulation of PSs in normal tissues/cells may induce side effects. Fortunately, the liposome membrane provides numerous immobilized sites for recognition moieties, such as antibodies, ligands, peptides,^[^ ^106^ ^]^ and electric charges. Additionally, biomimic liposomes fabricated from cracked cancer cell membranes, extracellular vesicles (EVs), and red blood cell membranes have shown promising advantages owing to their intact materials (**Figure** **7**).

**Antibody‐conjugated liposomes**: Antibodies exhibit high specific recognition ability against antigens in complicated physiological environments (so‐called immune‐targeting). Many antibodies, such as antibodies against CD19,^[^ ^107^ ^]^ CD20,^[^ ^108^ ^]^ CD74,^[^ ^109^ ^]^ HIF‐1*α*,^[^ ^83^ ^]^ EGFR,^[^ ^110^ ^]^ and HER2^[^ ^111^ ^]^ have been selected for liposomal immune‐targeting (Figure 7A). HER2 antibody‐immobilized liposomes Her2‐I&D‐LSL were delivered specifically to HER2‐overexpressing MCF‐7 and SKOV3 cells instead of A549 cells whose HER2 receptor expression is low.

In addition, because the size of full‐length or partial antibodies (55−150 kDa) may interfere with the preparation and biodistributions of liposomes, a single domain antibody was alternatively conjugated onto liposomes containing PS (**ZnPc**) to enhance the specificity of delivery.^[^ ^112^ ^]^

**Aptamer‐conjugated liposomes**: Like antibodies, aptamers have shown excellent specificity and affinity to cellular targets and have been widely used in targeted drug delivery (Figure 7B).^[^ ^113^ ^]^ For instance, the nucleolin‐specific aptamer AS1411, a G‐rich DNA oligonucleotide, was modified on the surface of the liposome UCNPs@BPQDs@Apt‐Lip (UBAL), that encapsulated upconversion nanoparticles@black phosphorus quantum dots (UCNPs@BPQDs).^[^ ^114^ ^]^ The aptamer‐modified liposome guided the tumor cell‐targeted combined therapies of PTT and PDT.

**Ligand (peptide)‐conjugated liposomes**: Biomarkers, such as the folate receptor, transferrin receptor, integrin (*α*_v_*β*_3_), and CD44, which are overexpressed on cancer cells, have been utilized for tumor‐targeting liposomal delivery.^[^ ^115^ ^]^ (Figure 7C) The receptors on cancer cells promote the intracellular uptake of the liposome by receptor‐mediated endocytosis followed by drug release in the targeting cells. RGD (arginine‐glycine‐aspartic acid) peptide as a specific ligand for integrin (*α*_v_ *β*_3_) was incorporated into the lipid‐PEG motif, which was assembled into liposomes together with PS and other drugs.^[^ ^116^ ^]^ Thus, the targeted RGD‐SSL‐[P]‐[S] displayed specific toxicity to human umbilical vein endothelial cells (HUVECs) with neovascularization, whereas no obvious damage to normal ARPE‐19 cells was observed. Self‐assembled liposome‐porphyrin (Lip‐p‐OH) was modified with an HA derivative to target CD44‐overexpressed cancer cells, such as MDA‐MB‐231 cells.^[^ ^117^ ^]^

**Biomimic liposomes**: Compared with “artificial” methods to introduce recognition moieties on the surface of liposomes, the biomimic method to prepare “endogenous” liposomes for drug delivery has promising advantages.^[^ ^27^ ^]^ As its endogenous characteristics, the reassembly of natural cell membranes to liposomes as drug carriers has good biocompatibility and minimum immune‐resistance.

**Figure 8:**
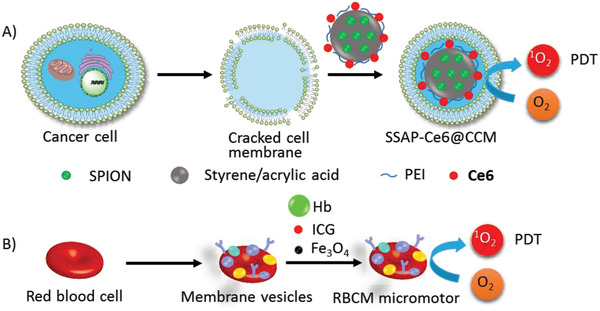
Biomimic liposomes made from A) cancer cell membrane, B) red blood cell membrane.

Compared with the components of conventional active‐targeted liposomes, the materials from cancer cells contain more complicated components, such as membrane enzymes, ligands, lipids, and carbohydrates. Therefore, cancer membrane reconstruction with liposomes possesses additional advantages such as activation of the immune system against cancer.^[^ ^118^ ^]^ Liposomes derived from homologous tumor cell membranes provided highly specific targeting by self‐recognition internalization in the delivery of nanoparticles into the targeted tumor (**Figure** **8**A).^[^ ^23^, ^119^ ^]^ Liposomes coated with cracked cancer cell membrane were also reported.^[^ ^120^ ^]^

A red blood cell‐mimicking micromotor was constructed using hemoglobin (Hb) particles containing magnetic Fe_3_O_4_ and **ICG** in a natural red blood cell membrane, which could actively deliver oxygen and PS to enhance PDT (Figure 8B).^[^ ^24^ ^]^ Because of the biconcave discoidal structure of red blood cells and higher density, RBCM (red blood cell membrane) micromotors could be driven by ultrasonic waves at a speed of up to 56.5 µm s^−1^ in biological media, and the motion can be directed by an external magnetic field. The formulation had good biocompatibility and stability in blood.

#### 2.3.2 Activatable Liposomes for Cargos Release

Due to their special structure, liposomes can encapsulate hydrophobic or hydrophilic materials, including dyes for imaging, chemotherapeutic drugs for chemotherapy, and genes for gene therapy. Drug delivery by conventional liposomes mainly depends on the EPR effect. However, the site specificity of drug delivery and release is not satisfactory for pursuing better therapeutic effects and fewer side effects. On the platform of liposomes, tumor‐targeting drug release has been successfully achieved by endogenous stimuli (pH and enzyme) and controllable external stimuli (heat, light, and X‐ray).^[^ ^121^ ^]^

**Figure 9:**
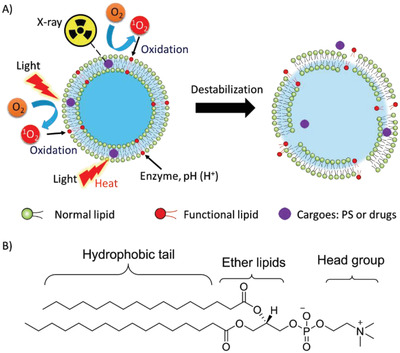
A) Liposomal collapse induced by various stimuli, such as light, photothermal effect, enzyme, pH, and X‐ray. B) Typical modification sites on lipid backbone for liposomal activation.

For liposomes comprising natural lipids or similar components, modifications were made to the backbone of lipids to induce the destabilization of liposomes and triggered the cargo release upon changes in lipid conformation (physical) or structure (chemical) in response to the various stimuli mentioned above (**Figure** **9**A). Three parts are available for modification: hydrophilic lipid head group, glycerol backbone, and hydrophobic fatty acid chain (Figure 9B).

**Figure 10:**
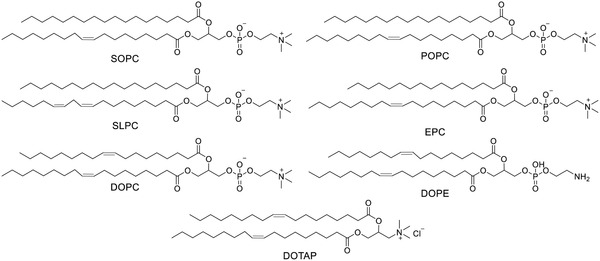
The structures of ROS‐sensitive lipids with C═C bond.

**Light‐sensitive liposomes**: Light is a non‐invasive, site‐controllable, and safe stimulus to remotely induce the destruction of liposomes and release of cargoes.^[^ ^122^ ^]^ The principles are mainly dependent on the molecular mechanism of photo‐triggerable lipid and subsequent destabilization of liposomes. ROS‐sensitive liposomes are disrupted by photo‐induced oxidation of unsaturated lipids to destabilize the liposomes to release the cargoes, including chemotherapeutic agents, fluorophores, imaging contrasts, and genes. As shown in **Figure** **10**, unsaturated fatty acids are responsible for the ROS‐sensitive moiety, such as SOPC,^[^ ^123^ ^]^ SLPC,^[^ ^123^ ^]^ DOPC,^[^ ^124^ ^]^ DOPE,^[^ ^125^ ^]^ DOTAP,^[^ ^126^ ^]^ POPC,^[^ ^127^ ^]^ and EPC^[^ ^82^ ^]^.

The photooxidation of phospholipids and the subsequent membrane degradation was investigated on the combination of unsaturated lipids (SOPC, SLPC, and DOPC) with PSs (**verteporfin**, **pheoA**, and **m‐THPP**) in photo‐triggerable liposomes.^[^ ^123^ ^]^ Particularly, m‐THPP/DOPC was the most efficient system for DOX release and has potential in chemotherapy and PDT.

ROS generated in light‐induced PDT oxidize unsaturated lipid (POPC), which induces controllable release of cargo (a fluorophore) from liposomes.^[^ ^127^ ^]^ Comparing two phenothiazinium dyes, **MB** and **DO15**, the research further suggested that direct physical contact with lipid double bonds improved the oxidation, which is noteworthy for optimizing the combination of activatable lipids and PSs for ROS‐sensitive liposomes.

The tissue penetration of cargoes released on demand depends on the efficiency of the activation of PS by light. Due to the tissue absorption, NIR has better penetration than ultraviolet and visible light. For example, a ROS‐responsive liposome GDPPL, fabricated with **DSPE‐PEG‐PheoA**, DPPC, DOPE, and other components was triggered by NIR irradiation (670 nm) to release the chemotherapeutic drug gemcitabine (GEM).^[^ ^125^ ^]^ NIR (808 nm) irradiation also activated liposomes containing lipid egg‐yolk L‐phosphatidylcholine (EPC), PS cypate (Cy), and other drugs.^[^ ^105^ ^]^

Compared with visible light and NIR, X‐rays have unlimited tissue‐penetration in medical examination and therapy, such as X‐ray checking, CT, and radiotherapy. Recently, in an X‐ray‐triggerable liposome, X‐ray activated PSs (**verteporfin** and **gold nanoparticles**) to produce singlet oxygen, which destabilized the liposome by oxidizing unsaturated lipid DOTAP to release a gene silencing reagent (antisense oligonucleotide) or a chemotherapeutic drug (doxorubicin, DOX).^[^ ^126^ ^]^ The system was also modified by triphenylphosphine (TPP) for mitochondria‐targeted delivery.^[^ ^128^ ^]^

**Thermo‐sensitive liposomes** are activated by photothermal effects, where liposomes are self‐assembled by lipids and other additives depending on the stability of lipids under the lipid phase transition temperature. This means that liposomes will be destabilized and release cargoes at the desired site once the actual temperature is higher than the phase transition temperature by controlled heating.^[^ ^129^ ^]^ Therefore, the selection of lipids with a phase transition temperature just above the human physiological temperature (37 °C) is a key factor for the construction of thermo‐sensitive liposomes. Another factor is the photothermal agent that efficiently converts light energy into heat. Thermo‐sensitive liposomes encapsulated with **ICG** as a photothermal agent were triggered by NIR irradiation to release cargos, where DPPC as one of the major components has a phase transition temperature of 41 °C.^[^ ^130^ ^]^ Thermo‐sensitive liposomes are often involved in the combined therapy of PTT with PDT, chemotherapy, and so on.

**pH‐responsive liposomes**: In the tumor physiological environment, pH values range from 5 to 6.3, which is different from the normal physiological pH (7.0−7.4). pH is often used to activate pH‐sensitive molecules/materials, such as lipids^[^ ^83^ ^]^ and peptides,^[^ ^131^ ^]^ to induce site‐specific liposomal drug release. In a pH‐sensitive liposome encapsulated PS (**DiBDP**), the lipid DOPE is sensitive to pH.^[^ ^83^ ^]^ At a physiological pH of 7.4, **DiBDP** was slowly released, i.e., only 9.1% of **DiBDP** was released over 24 h of incubation. However, this was significantly accelerated at pH 6.0, owing to the pH‐induced degradation of DOPE in the Ab‐DiBDP NPs. The fastest release of **DiBDP** was observed at pH 5.0, reaching a plateau at ≈86% within 24 h. Moreover, a smart liposome was fabricated using the acid‐responsive phosphatidylethanolamine poly(L‐histidine)_40_(PE‐p(His)_40_) and temperature‐responsive FA‐conjugated PE‐p(NIPAM) (PE‐p(NIPAM)40‐FA).^[^ ^132^ ^]^ The swelling of the histidine block, where the imidazole was ionized under acidic conditions, was responsive to drug release.

Other pH‐sensitive nanoparticles also facilitate construction of hybrid nano‐platforms joined with liposomes for acid‐responsive drug release. For example, zinc oxide (ZnO) as an acid‐responsive material has been incorporated into liposomes for drug delivery in cancer therapy.,^[^ ^133^ ^]^ The hybrid nanocomplexes released the chemotherapeutic drug (DOX) more rapidly than bare liposomes, thus enhancing the therapeutic effects upon exposure to the acidic physiological environment in cancer cells. In other study, pH‐sensitive CaP played a similar role in drug release.^[^ ^134^ ^]^

## 3 Biomedical Applications of Liposomal System in Tumor Theranostic

After the illustrations of the single element of the principles of liposome‐based drug delivery, such as liposomal PSs, targeting models of liposomes and triggerable release models of liposomes, the multi‐function of liposomes were introduced to synergistically enhance PDT. To address the limitations of PDT monotherapy, co‐capsulated drugs have been developed for example, TME for therapy resistance, including hypoxia and tumor extracellular matrix. Moreover, the solutions to enhance the specificity of delivery and release may reduce the dose and side effects. Therefore, multi‐functional liposomes in response to environmental stimuli for combined diagnosis/therapy with PDT have emerged rapidly as a new tendency in applications, including image‐guided PDT, PDT/chemotherapy, PTT/PDT and etc., since Zheng et al. fabricated an excellent example of multi‐functional nano‐platform based on liposomes called “porphysomes” in 2011.^[^ ^135^ ^]^

### 3.1 Enhancing Tissue‐Penetration of PDT

Tissue penetration is one of the main challenges in tumor PDT, particularly in PS delivery and light irradiation. On one hand, the challenge of PS delivery can be addressed by liposome‐guided delivery, as summarized in the previous Section 2.3.

**Figure 11:**
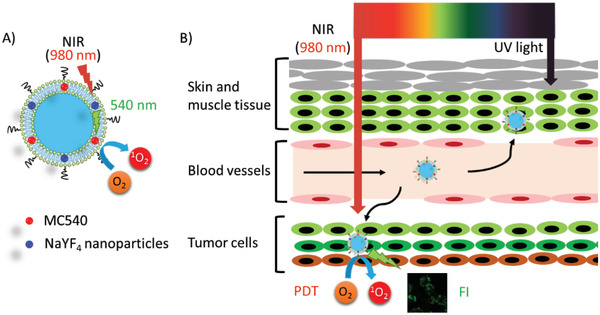
A) NIR irradiation was converted to visible light (540 nm) by NaYF_4_ UCN to activate liposomal PS (**MC540**) for B) PDT and FLI. Reproduced with permission.^[^ ^137^ ^]^ Copyright 2014, American Chemical Society.

On the other hand, light penetration largely depends on its wavelength due to tissue absorption. For a single PS, longer wavelength of exciting light leads to better tissue penetration for activating PDT. In addition to developing new PSs with longer wavelengths of absorption toward the NIR window, in situ light‐generating strategies for PS activation have been investigated to overcome the limited penetration of visible light. For example, a flexible strategy based on the liposome system was reported by Bonnet et al. and Chang et al., respectively. In a proof‐of‐concept study by Bonnet et al.,^[^ ^136^ ^]^ PS (**ruthenium complex**) was activated by the blue light transformed from light at 532 or 630 nm, respectively, through triplet‐triplet annihilation upconversion (TTA‐UC). Thereafter, Chang et al. made significant progress in a polymeric liposome co‐loaded with PS (Merocyanine 540, **MC540**) and up‐conversion nanoparticles (UCNs) for PDT and FLI (**Figure** **11**).^[^ ^137^ ^]^ UCNs converted NIR (980 nm) to visible light (540 nm) to activate PS (**MC 540**) in depth. In another liposomal system (Hb‐NPs@liposome), in situ luminescence catalyzed by Hb in the presence of luminol and endogenous H_2_O_2_, overcome the limitation of light penetration.^[^ ^138^ ^]^ Radiolabeled Europium‐loaded liposomes (^64^Cu‐Eu/VBBO lipo) emit radioluminescence (red fluorescence) to activate PS.^[^ ^139^ ^]^

Unlike the typical PDT working with three essential elements (light, PS, and oxygen), X‐ray‐induced PDT has overcome the reliance on light, which has excellent tissue penetration ability compared with only up to 3 mm penetration of red light (620–750 nm) required by **verteporfin** activation.^[^ ^128^ ^]^ The mitochondria‐targeted liposomes (TPP‐Lipo‐VP‐10Au) encapsulated PS **verteporfin** and gold nanoparticles. The results indicated that ^1^O_2_ generation was enhanced by ≈129% and 186% by gold nanoparticles with diameters of 5 and 10 nm, respectively, in the presence of X‐rays (4 Gy).

### 3.2 Enhancing PDT Against TME

The TME is highly associated with tumor progression, intractable metastasis, and especially resistance to therapies. The TME is characterized by the typical features of tumors, such as hypoxia, oxidative stress, and acidosis, and induce changes in the components of the ECM, immune response, and angiogenesis. Therefore, new therapeutic strategies targeting the TME have become essential to overcome tumor resistance.^[^ ^8^, ^140^ ^]^

#### 3.2.1 Hypoxia‐Relief

**Figure 12:**
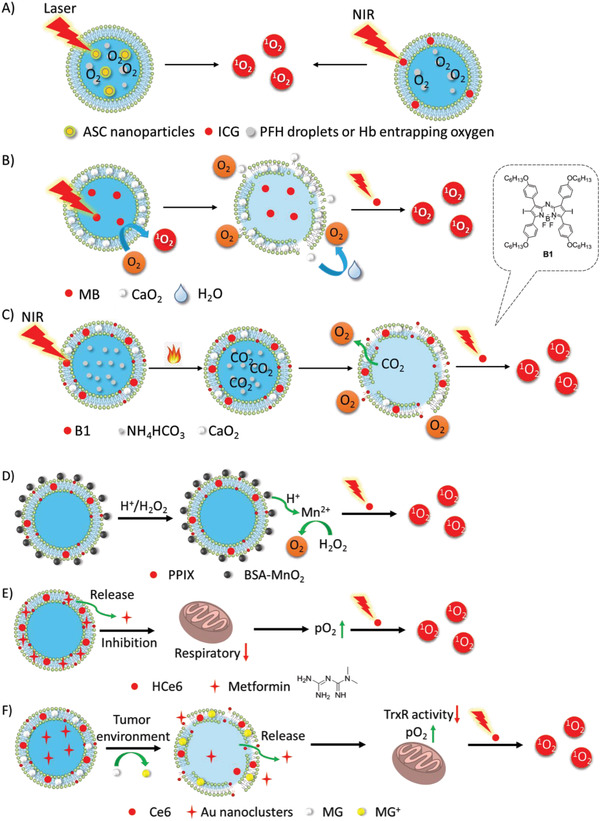
Three ways to enhance the supply of cellular oxygen to PS: A) delivery of extracellular oxygen by oxygen carriers (PTF and Hb); B,C) delivery of oxygen‐generating materials (CaO_2_) to PS; D) transformation of other endogenous molecules (H_2_O_2_) to oxygen; E,F) suppression of other pathways that consuming intracellular oxygen by inhibitors (metformin, Au nanoclusters).

As PDT is an oxygen‐dependent procedure, it can be suppressed by hypoxia regardless of PDT‐induced hypoxia or tumor hypoxia. In order to improve PDT efficiency under hypoxia, there are three ways to enhance the supply of cellular oxygen to PS: 1) delivery of extracellular oxygen or oxygen‐generating materials to PS, 2) transformation of other endogenous molecules to oxygen, and 3) suppression of other pathways consuming intracellular oxygen (**Figure** **12**).

Since 2000, the delivery of exogenous oxygen to tumors by hyperbaric oxygen has been reported to enhance PDT in advanced carcinoma in the clinical use.^[^ ^141^ ^]^ However, the delivery relies heavily on blood transport, which may be blocked by the ablation of tumor blood vessels in PDT, thereby blocking accessibility to the tumor interior. Some oxygen‐carrying and oxygen‐generating materials, including perfluorocarbon compounds,^[^ ^142^ ^]^ Hb, red blood cells, and CaO_2_, efficiently relieve hypoxia to enhance PDT. Perfluorohexane (PFH) droplets entrapping oxygen were encapsulated in a liposomal system (Lip(ASC/PFH) containing nanoparticle Au@SiO_2_@Cu_2_O (ASC) as PS for enhancing tumor PDT (Figure 12A).^[^ ^143^ ^]^ Besides, as tissue hypoxia is also a common microenvironmental feature of bacterial infection‐induced inflammation, new approaches to anti‐infective therapeutics have to overcome this problem.^[^ ^144^ ^]^ PFH was integrated into a PFH/IR780@liposome coating on titanium (Ti) for antibacterial PDT.^[^ ^145^ ^]^ After only 15 min, the antibacterial efficacy against *Escherichia coli* and *Staphylococcus aureus* was improved from 66.54% and 48.04%, to 99.62% and 99.63%, respectively. Hb, an oxygen‐carrying protein in red blood cells, is an ideal oxygen donor. Thus, liposomal oxygen‐saturated Hb efficiently alleviated tumor hypoxia to enhance PDT (Figure 12A).^[^ ^138^, ^146^ ^]^ Upon NIR irradiation, liposomal ICG co‐encapsulated with Hb showed greater accumulation in tumors and higher antitumor activity. Calcium peroxide (CaO_2_) as an oxygen‐generating material and MB were encapsulated into an oxygen‐self‐supplying liposome‐based nanoparticle, LipoMB/CaO_2_ (Figure 12B).^[^ ^147^ ^]^ Upon light irradiation, triggered by oxidative degradation of lipids by ROS, CaO_2_ was released from the liposome to react with water to produce oxygen for PDT, which efficiently relieved tumor hypoxia. In the liposome (CaO_2_/B1/NH_4_HCO_3_ lipo), aza‐BODIPY dye (**B1**), and CaO_2_ nanoparticles were encapsulated in the hydrophobic layer and NH_4_HCO_3_ was encapsulated in the hydrophilic cavity (Figure 12C).^[^ ^148^ ^]^ Upon NIR irradiation, **B1** was activated to induce hyperthermia and further triggered the decomposition of NH_4_HCO_3_. Subsequently, with the aid of NH_4_HCO_3_ and CaO_2_ nanoparticles, oxygen was rapidly and self‐sufficiently generated to relieve hypoxia, during which clean by‐products were produced.

Endogenous H_2_O_2_ generated in the mitochondrion could be an additional source of cellular oxygen. In PPIX‐Lipo‐M, the in situ production of oxygen from endogenous H_2_O_2_ was catalyzed by MnO_2_ to relieve hypoxia (Figure 12D).^[^ ^149^ ^]^ With good biocompatibility and high catalytic capacity, liposomal catalase also catalyzed the transformation of H_2_O_2_ to O_2_ to alleviate tumor hypoxia, which was validated to enhance not only PDT, but also antitumor immunity by reversing the immunosuppressive TME.^[^ ^150^ ^]^ The contribution of the latter to enhance PDT has often been neglected in most studies on hypoxia‐relief strategies. Furthermore, the generation of endogenous H_2_O_2_ was promoted by adjusting the redox equilibria of tumor cells upon GSH consumption, which was converted to O_2_ and ROS for type II and type I PDT by Fenton reaction, respectively.^[^ ^105^ ^]^

Several strategies have been reported to suppress oxygen consumption during respiration, thus increasing the oxygen supply to PDT. Liposomal metformin which inhibits mitochondrial respiration, relieved tumor hypoxia to enhance PDT (Figure 12E).^[^ ^151^ ^]^ Liposomal gold (Au) nanoclusters inhibited thioredoxin reductase (TrxR) to enhance tumor PDT (Figure 12F).^[^ ^152^ ^]^ After the liposome was internalized specifically by tumor cells into the acidic lysosome via antibody HER2, malachite green carbinol base (MG), a pH responder anchored on the liposome membrane, was converted to MG carbocation (MG^+^), leading to liposome collapse and the payload release of Au nanoclusters and **Ce6**. Subsequently, the Au nanoparticles inhibited TrxR activity to provide more oxygen supply for the enhancement of PDT.

#### 3.2.2 GSH Synthesis Antagonist

**Figure 13:**
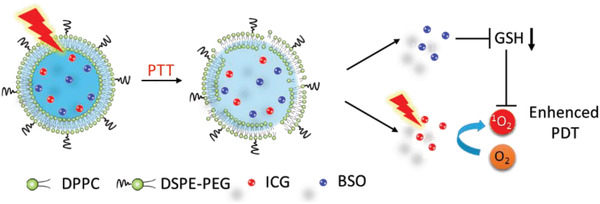
Proposed mechanism of PLNA for enhanced PDT via NIR remote ‐controlled BSO release for the inhibition of GSH biosynthesis.

In cancer cells, excessive concentration of GSH as a ROS scavenger compromises the efficiency of PDT. Therefore, NIR‐photothermal liposomal nanoantagonist amplified cancer PDT (**Figure** **13**).^[^ ^153^ ^]^ L‐Buthionine sulfoximine (BSO), a GSH synthesis antagonist, was encapsulated in a thermally responsive liposome (PLNA) and released by NIR irradiation.

#### 3.2.3 Tumor ECM

**Figure 14:**
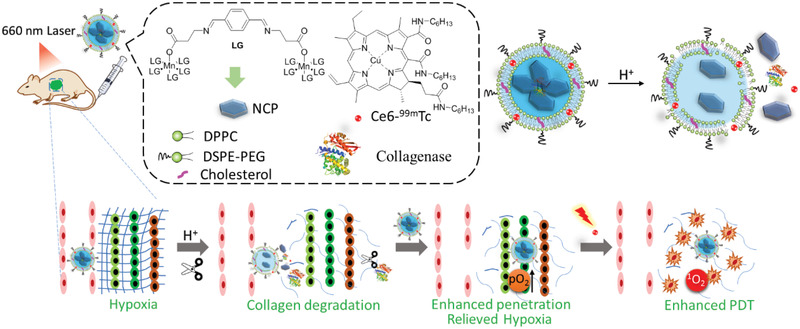
A schematic illustration of the components of CLG@NCP‐PEG and the mechanism of its pH‐sensitive degradation and collagen degradation to enhance PDT.

The abundant tumor ECM results in insufficient tumor retention and ineffective intra‐tumor penetration of therapeutic agents, as well as acidity and hypoxia of TME, further leading to unsatisfactory therapeutic outcomes for many therapies. Collagenase (CLG) was encapsulated into acid‐responsive nanoparticles (NCPs) and then delivered in liposomes co‐loaded with **Ce6‐^99m^Tc** (**Figure** **14**).^[^ ^154^ ^]^ The released collagenase degraded collagen (a major component of ECM) to facilitate the penetration of **Ce6** and relieve hypoxia to enhance PDT. In another study, liposomal formation of hydrophobic **P18** enabled ECM destruction and facilitated the chemotherapy by Taxol.^[^ ^60^ ^]^

### 3.3 Image‐Guided PDT

**Figure 15:**
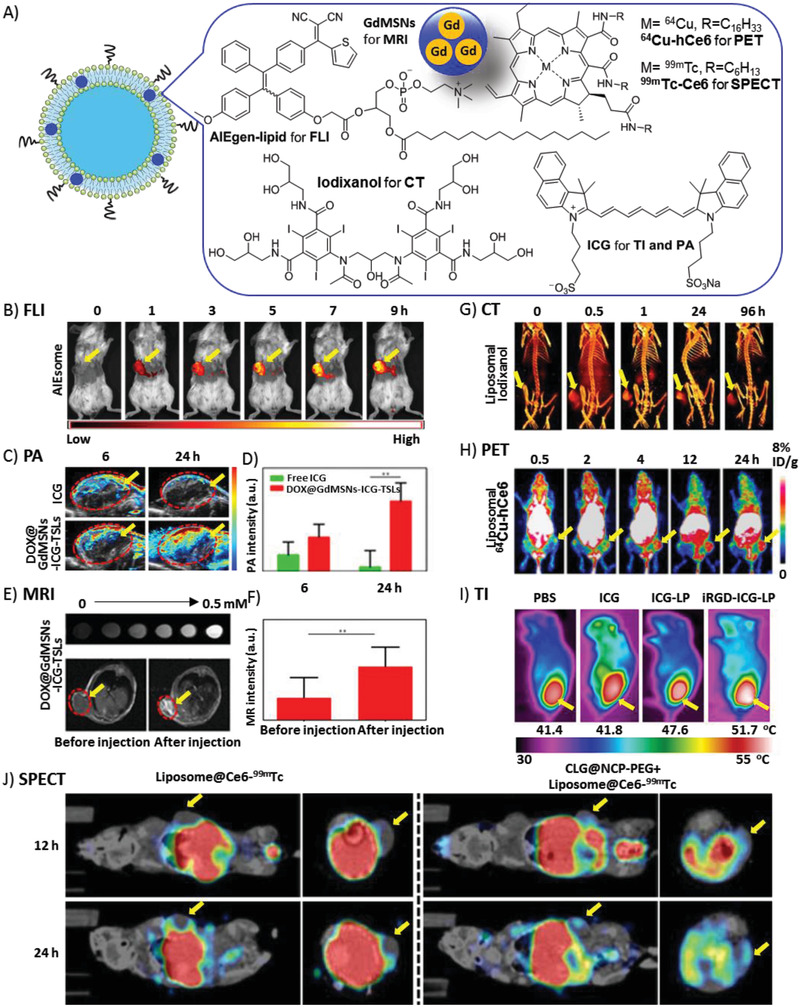
A) Liposomes encapsulating various imaging agents for visualization of tumor‐bearing mice. Tumors are indicated by yellow arrows. B) FLI after i.v. injection of AIEsomes. Reproduced with permission.^[^ ^93^ ^]^ Copyright 2018, WILEY VCH GmbH & Co. KGaA. C) PA images after i.v. injection of free **ICG** and DOX@GdMSNs‐ICG‐TSLs. D) PA intensity of tumor sites after treatment with free **ICG** and DOX@GdMSNs‐ICG‐TSLs. E) T1‐weighted MR images of DOX@GdMSNs‐ICG‐TSLs nanoparticles at various Gd concentrations (top). T1‐weighted MR images before and after injection with DOX@GdMSNs‐ICG‐TSLs (bottom). F) Relative MR intensities before and after the injection of DOX@GdMSNs‐ICG‐TSLs. C‐F) Reproduced with permission.^[^ ^30^ ^]^ Copyright 2018, American Chemical Society. G) 3D volume‐rendered images after injection with NL‐co‐encapsulated iodixanol and **TPPS_4_** (LIT). Reproducedunder the terms of the Creative Commons CC‐BY license.^[^ ^58^ ^]^ Copyright 2019, Ivyspring International Publisher. H) In vivo PET images after i.v. injection of ^64^Cu^2+^‐labeled AQ4N‐hCe6‐liposome. Reproduced with permission.^[^ ^160^ ^]^ Copyright 2017, American Chemical Society. I) Infrared photothermal images after i.v. injection of liposomal **ICG** followed by laser irradiation. Reproduced with permission.^[^ ^161^ ^]^ Copyright 2015, Elsevier. J) In vivo SPECT imaging after i.v. injection of Liposome@Ce6‐^99m^Tc. Mice with (right) and without (left) pretreatment of CLG@NCP‐PEG were visualized. Reproduced with permission.^[^ ^154^ ^]^ Copyright 2018, American Chemical Society.

The intrinsic features of tumors at every stage make diagnosis very difficult. For instance, the infiltrating cancer lacks a clear boundary with the surrounding normal tissues. Advanced and metastatic cancer complicates therapy, as single tumor cells and tumor tissue are too small to be detected. Liposomes exhibit a large capacity for the co‐encapsulation of reagents for medical imaging. Recently, Chen et al. reviewed liposome‐based probes for molecular imaging, including photoacoustic (PA) imaging, computed tomography (CT), magnetic resonance imaging (MRI), ultrasonic imaging (USI), positron emission tomography (PET) imaging, and FLI.^[^ ^155^ ^]^ Herein, we focused on liposome‐based imaging, which provides direct and visual evidence of the delivery, accumulation, and metabolism of the functional liposomes in vivo. These studies guide PDT protocols and those of other combined therapies, and evaluate the therapeutic efficiencies (**Figure** **15**).

#### 3.3.1 FLI

FLI is a highly sensitive, real‐time and low‐cost characterization tool for cell‐based drug discovery and image‐guided therapy. FLI provides useful information about the bio‐distribution of PS in organs and tumors, and therapeutic effects by estimation of tumor size. On the one hand, the dual‐functional drugs, such as AIEsome,^[^ ^93^ ^]^ act as a PS and fluorophore in PDT, which is simultaneously serviced in PDT and FLI of tumors (Figure 15A,B). On the other hand, although most PSs can be used as fluorescent dyes, the wavelength of fluorescent emission in the visible region has limited tissue penetration. To address this, other NIR dyes, such as IRDye800CW,^[^ ^156^ ^]^ has been co‐encapsulated in liposomes. However, the applications of FLI remained to be limited by poor tissue penetration, even when NIR is applied.

#### 3.3.2 PA Imaging

PA imaging has shown promising advantages in in biomedical imaging, including deep tissue imaging, ROS detection, and therapeutic response detection, due to its non‐toxicity, non‐invasion, good penetration, real time, and high spatial resolution, in comparison with conventional imaging techniques.^[^ ^157^ ^]^ PA is based on the PA effect by converting the light energy absorbed into thermal energy and acoustic energy. Liposomal PA contrast agents, as well as photothermal agents, include **ICG** ^[^ ^102^ ^]^ and fullerene derivative **C2a**.^[^ ^158^ ^]^ PA showed that liposomal formation of **ICG** had much more accumulation and longer retention time in the tumor than free **ICG** (Figure 15A,C,D).^[^ ^30^ ^]^ Therefore, PA imaging is capable of monitoring the bio‐distribution of co‐capsulated liposomal PS and its therapeutic effects in PDT.

#### 3.3.3 MRI

MRI is a popular non‐invasive imaging technique with high temporal and spatial resolution, and used in clinical medical checking. MRI detects the protons of water and fat molecules in soft tissues and blood circulation. However, to achieve higher sensitivity, additional contrast agents are often needed for intravenous injection. In a hybrid theranostic nano‐platform, DOX@GdMSNs‐ICG‐TSLs, gadolinium (Gd), a T1 magnetic MRI contrast agent, was doped into MSNs, which were encapsulated into liposomes to guide synergistic tumor therapies.^[^ ^30^ ^]^ (Figure 15A,E,F) After calibration, MRI revealed the high accumulation of the nanocomposite in the tumor, suggesting that liposomal Gd is a safe and effective contrast agent. However, MRI is not suitable for the human bodies with magnetic metal implants, such as heart pacemakers and artificial teeth.

#### 3.3.4 CT

CT has high 3D resolution of soft tissue and excellent tissue penetration. It has been widely used for the diagnosis of diseases before and after treatment. An FDA‐approved contrast agent (iodixanol) was encapsulated into the nanoliposome for CT together with PS (**TPPS_4_**) for PDT (Figure 15A–G).^[^ ^58^ ^]^ The liposomal theranostic platform of X‐ray CT/FLI‐guided PDT has shown potential in clinical use because of its high spatial resolution and high sensitivity. CT imaging clearly revealed the bio‐distribution of the liposomal contrast agent in the kidney, bladder, and tumor. The bimodal imaging showed that, in tumor PDT in vivo, the efficiency of the liposomal formulation of **TPPS_4_** was much higher than that of other formulations. However, CT has a limitation in the dosage of radioactive X‐rays.

#### 3.3.5 PET Imaging

PET imaging has shown promising advantages in bio‐imaging and medical examinations as it has high sensitivity, high spatial resolution, excellent tissue penetration, and is capable of dynamic real‐time checking. Compared with CT and MRI, PET imaging displays metabolic activities, which can distinguish tumors from normal tissues. Isotopes with a suitable half‐life that emit positrons are key in PET imaging. Cancer cell membrane‐derived liposomes bearing ^89^Zr as a PET tracer and **tetrakis(4‐carboxyphenyl) porphyrin** as PS, were successfully applied in theranostic for 4T1 tumors.^[^ ^159^ ^]^ Interestingly, PET imaging showed that ^89^Zr‐Df‐MCLs efficiently accumulated in the tumor. Furthermore, another PET tracer ^64^Cu was chelated with PS **Ce6** to give AQ4N‐^64^Cu‐hCe6‐liposome, which was responsible for PET, PA, and FL imaging (Figure 15A–H).^[^ ^160^ ^]^ As the long half‐life of ^64^Cu (*T*_½_ = 12.7 h), it clearly and quantitatively demonstrated the pharmacokinetic and time‐dependent bio‐distributions of ^64^Cu‐labeled liposomes in organs and tumors of mice in real‐time of up to 24 h. However, PET imaging is limited by the dosage of ionizing radiation.

#### 3.3.6 SPECT Imaging

Single‐photon emission computerized tomography (SPECT) is widely used in diagnostic clinical studies owing to its high tissue‐penetration ability, high sensitivity, and high spatial resolution. Similar to PET, SPECT adopts isotopes emitting *γ*‐rays, such as ^99m^Tc with a half‐life of ≈6 h. In ^99m^Tc^4+^‐labeled liposome@Ce6 (Liposome@Ce6‐^99m^Tc), **Ce6‐^99m^T** was used as the tracer, and PS was incorporated (Figure 15J).^[^ ^154^ ^]^ The biodistribution of the liposomes was quantitatively detected, showing that CLG efficiently improved the delivery to the tumor interior by the degradation of collagen among tumor cells. However, SPECT imaging carries the risk of exposure to ionizing radiation.

#### 3.3.7 Thermal Imaging (TI)

TI is a noninvasive, non‐radioactive, and convenient tool for monitoring body temperature abnormalities‐related diseases, especially for early diagnosis of tumor and blood vessel damage. For the liposomal nano‐platform for combination of PDT and PTT, TI revealed the biodistribution of liposomes containing **ICG** as a PS and photothermal agent, as well as the disruption of photothermal‐responsive liposomes during drug release (Figure 15I).^[^ ^161^ ^]^ However, TI has poor tissue penetration and low resolution.

### 3.4 Synergistic Combination of PDT with Other Therapies

**Figure 16:**
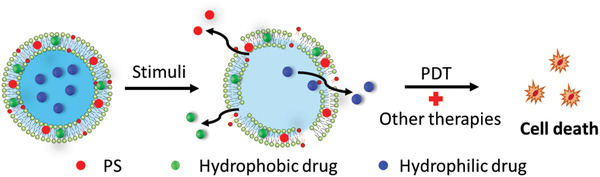
The mechanism of release of capsulated PSs and drugs for combined therapies.

PDT by itself has limitations in oxygen‐dependence, poor tissue‐specific delivery of PS and poor tissue penetration, and other therapies have their own limitations as well. By the synergism of different therapies via different mechanisms in tumor‐cell killing, it is possible to minimize the resistance to monotherapy, doses of therapies, and therefore the side effects to normal tissues, while achieving the same level in tumor suppression as monotherapy (**Figure** **16**).

#### 3.4.1 PDT with Cytotoxic Agents

Cytotoxic agents, especially chemotherapeutic agents, have distinct advantages in killing tumor cells and preventing tumor reoccurrence systematically by inhibiting the survival and metastatic pathways of targeted tissues/cells. However, they also suffer from poor stability in physiological media, poor tumor cell specificity, and severe side effects due to exposure to normal tissue/organ. Liposomes efficiently resolve these limitations to improve therapy (**Table** **2**).

**Table 2:** Representative cytotoxic agents co‐encapsulated with liposomal PSs

Entry	Prodrug/Drug	Release mode of liposome	Mechanism	Cell lines/tumor	Ref.
1	Doxorubicin (DOX)	ROS‐, pH‐sensitive	DNA damage and EGFR/Src/HMG‐CR pathway inhibition	MIA PaCa‐2, HeLa, PDAC, MCF‐7, SKOV3, A549 cells	^[^^111^, ^124^, ^134^, ^167^, ^168^^]^
2	Cabzantinib(XL184)	ROS‐sensitive	Multikinase inhibitor	AsPC1 cells	^[^ ^170^ ^]^
3	Lapatinib	–	EGFR inhibition	Glioma cell lines	^[^ ^171^ ^]^
4	Lonidamine	Thermo‐sensitive	Inhibiting hexokinase and acting on mitochondrial adenine nucleotide translocase	LL/2 cells	^[^ ^172^ ^]^
5	17‐AAG	Thermo‐sensitive	HSP90 inhibitor; Suppress pro‐survival and angiogenic signaling subunits	SCC‐7 and MCF‐7 cells	^[^ ^173^ ^]^
6	Cisplatin	–	Inhibiting DNA via DNA‐crosslinking	MPNST Cells, S462‐TY Xenograft Tumor	^[^ ^174^ ^]^
7	HOC	Thermo‐sensitive	Anti‐Proliferation	4T1 cells	^[^ ^175^ ^]^
8	Paclitaxe(PTX)	ROS‐sensitive	Anti‐tublin	MCF‐7 cells	^[^ ^176^ ^]^
9	Acriflavine(ACF)	–	HIF‐1 inhibitor and potential dual topoisomerase I/II inhibitor,	SK‐ChA‐1 and A431 cells	^[^ ^177^ ^]^
10	GEM	ROS‐sensitive	Inhibit DNA synthesis	HuCCt‐1 cells	^[^ ^125^ ^]^
11	Tirapazamine (TPZ)	ROS‐sensitive	Break DNA and induce apoptosis	MCF‐7, 4T1, A431 and Sk‐Cha1 cells	^[^ ^178^ ^]^
12	AQ4N	–	The reduced product AQ4 inhibits topoisomerase II and binds tightly to DNA.^[^ ^162^ ^]^	4T1 cells	^[^ ^160^ ^]^
13	Bevacizumab	–	Antibody against VEGF	PDAC cells/tumor	^[^ ^179^ ^]^
14	TuBB‐9	–	Antibody against Ki‐67	HeLa cells	^[^ ^180^ ^]^
15	NLG919	–	Indoleamine‐2,3‐dioxygenase (IDO) inhibitor	4T1 cells/tumor	^[^ ^181^ ^]^
16	Saprin	ROS‐sensitive	Ribosome‐inactivating protein	MC28 fibrosarcom cells	^[^ ^182^ ^]^
17	D‐(KLAKLAK)_2_	D‐(KLAKLAK)_2_‐induced lysis and ROS oxidation	Mitochondria‐dependent apoptosis	KB cells	^[^ ^183^ ^]^
18	Miltefosine	–	Inhibits phospholipid and sterol biosynthesis and interferes with cell signal‐transduction pathways^[^ ^163^ ^]^	*Leishmania (L.) amazonensis*	^[^ ^184^ ^]^

As the first approved liposomal anticancer drug, DOX is widely used against numerous malignancies^[^ ^164^ ^]^ due to DNA damage and other mechanisms, such as EGFR/Src/HMG‐CR pathway inhibition.^[^ ^165^ ^]^ Because liposomal formulations have improved DOX‐related toxicities, such as cardiomyopathy,^[^ ^166^ ^]^ liposomal DOX has been co‐encapsulated with various PSs for combined therapies (Table 2, entry 1). For instance, dual‐functional liposomes encapsulated with DOX and **Ce6** enhanced tumor therapy.^[^ ^167^ ^]^ Porphyrin‐phospholipid (**PoP**) liposomes were triggered by NIR irradiation to rapidly release DOX.^[^ ^124^ ^]^ the oxidations of DOPC and cholesterol by ROS were responsive to drug release. DOX/ZnPc co‐loaded MSNs@CaP@PEGylated liposomes achieved a combination of PDT and chemotherapy for tumors, where CaP was pH‐responsive for DOX release.^[^ ^134^ ^]^ More details on the pharmacokinetics and pharmacodynamics of liposomal chemophototherapy were studied in short drug‐light intervals.^[^ ^168^ ^]^ The study revealed that better penetration and accumulation resulted in an overall 7.4‐fold increase in the DOX concentration in the tumor. You et al. combined photodynamic and chemotherapy, and study DOX release from ROS‐sensitive liposomes after NIR light irradiation of PS **ICG**.^[^ ^111^ ^]^ At the same time, the fluorescent emission of DOX (Ex/Em: 498/553 nm) facilitated the study of drug delivery and distribution by optical imaging.

Kinase inhibitors are very important anti‐cancer drugs for targeted therapy.^[^ ^169^ ^]^ The multikinase inhibitor cabozantinib (XL184) was released from ROS‐sensitive liposomes upon PDT, and then efficiently suppressed the resistance of tumor to PDT (Table 2, entry 2).^[^ ^170^ ^]^ First, XL184 inhibited the signaling transduction to promote cell survival against PDT damage. Second, XL184 inhibited vascular endothelial growth factor (VEGF) signaling pathway, promoting the recovery of tumor and vessel against PDT damage. Third, XL184 inhibited the MET (a receptor tyrosine kinase) signaling transduction to suppress tumor metastasis in response to hypoxia. The clinical epidermal growth factor receptor (EGFR) inhibitor lapatinib was also applied in liposome‐based PDT (Table 2, entry 3).^[^ ^171^ ^]^

The anti‐cancer drug lonidamine acting on mitochondria was co‐encapsulated in a mitochondria‐targeting liposomal system with NIR PS (IR‐780) for PTT and PDT (Table 2, entry 4).^[^ ^172^ ^]^ The cationic TPP was modified on liposome surface to achieve mitochondria‐selective accumulation. In addition, anti‐cancer agent tanespimycin (17‐AAG) and IR 820 were combined for synergistic phototherapy and chemotherapy (Table 2, entry 5).^[^ ^173^ ^]^ Chemotherapy reagent was released from the collapsed thermo‐sensitive liposomes by photothermal effect.

Platinum‐containing compounds, such as cisplatin (cDDP) and OXA, have been used as chemotherapeutic drugs for anti‐tumor. cDDP is a first‐line chemotherapeutic drug that inhibits DNA via DNA‐crosslinking. A dual‐effect liposome (PL‐CDDP‐Ce6) significantly improved tumor therapeutic efficacy while reducing its toxicity (Table 2, entry 6).^[^ ^174^ ^]^ Li et al. constructed a multi‐sensitive liposome (ELTSL) responsive to enzyme, light, and temperature for controllable release of PS and the chemotherapeutic agent HOC (Table 2, entry 7).^[^ ^175^ ^]^ Peptide‐polyethylene glycol (PEG) corona incorporated on the surface of liposomes is cleaved specifically by MMP‐2 overexpressed on tumor to enhance tumor penetration and cellular uptake of the liposome. Under NIR laser irradiation, **PPa** is not only a PS for PDT, but also a fluorophore for imaging and hyperthermia‐induced drug release of the lipophilic prodrug HOC and water‐soluble DOX. The unprotected Pt (IV) prodrug HOC was reduced to OXA (II) by endogenous GSH. This prodrug strategy improved the therapeutic outcomes of platinum drugs, while suppressing the side effects.

The anti‐Tublin inhibitor paclitaxel (PTX) was entrapped with PS sinoporphyrin sodium (**DVDMS**) by liposomal delivery to enhance singular therapy (Table 2, entry 8).^[^ ^176^ ^]^ This formulation of PTX prevented serious systemic toxicities and low selectivity.

Activation of downstream signaling transduction of HIF‐1 in human hilar cholangiocarcinomas and human epidermoid carcinoma (A431) cells contributes to the PDT‐resistance in post‐PDT survival. To overcome this resistance, HIF‐1 and topoisomerase were inhibited by liposomal acriflavine, to suppress cell survival against PDT based on liposomal **ZnPc** (Table 2, entry 9).^[^ ^177^ ^]^ Cationic liposomes facilitate tumor‐specific delivery.

The chemotherapeutic agent GEM not only induces drug resistance and system toxicity, but also easily is deactivated by metabolization. To enhance its tumor toxicity and reduce side effects, GEM was fabricated into a ROS‐responsive liposome GDPPL together with PS (**DSPE‐PEG‐PheoA**) (Table 2, entry 10).^[^ ^125^ ^]^ Lipid DOPE was responsive to ROS‐induced liposome collapse. Compared with that from non‐ROS activated liposomes, the release rate of GEM from ROS‐activated liposomes was faster by ≈2‐fold, thus providing better cytotoxicity to lesions. Furthermore, immune cells were recruited to the treated tumor.

**Figure 17:**
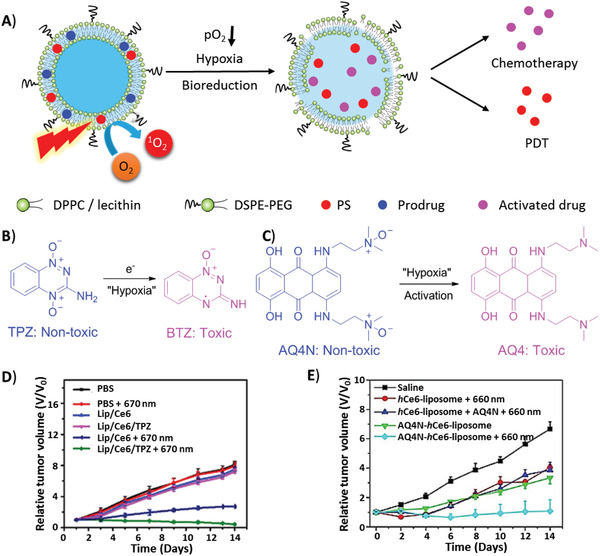
Combination of PDT and hypoxia‐activated chemotherapy. A) Prodrug liposomal TPZ and AQ4N were activated to toxic BTZ and AQ4 under hypoxia, respectively. B) Mechanism of transformation of TPZ and AQ4N under hypoxia. The relative tumor volume changes of mice treated with liposomes containing prodrug D) TPZ and E) AQ4N. D) Reproduced with permission.^[^ ^178b^ ^]^ Copyright 2018 Elsevier. E) Reproduced with permission.^[^ ^160^ ^]^ Copyright 2017 American Chemical Society.

Although liposome‐based combined PDT and traditional chemotherapy have shown good therapeutic effects, the risk of leakage of chemotherapeutic agents might induce potential side effects to normal tissues. A new strategy, particularly suitable for post‐PDT therapy, was developed to minimize side effects. When PDT is severely weakened by hypoxia, the toxicity of the chemotherapeutic prodrug is activated by hypoxia. The therapeutic prodrugs tirapazamine (TPZ)^[^ ^178^ ^]^ and AQ4N^[^ ^160^ ^]^ have been reported to combine with PDT, respectively (Table 2, entry 11 and 12) (**Figure** **17**). For example, after liposomes containing prodrug TPZ and PSs (**Ce6**, **IR780**, **ZnPc**, or **ICG**) were transported to the tumor, PDT induced the disruption of ROS‐responsive liposomes for drug release and consumption of the endogenous oxygen to exacerbate hypoxia. Subsequently, TPZ was activated to toxic BTZ by biogradation at the hypoxic site, which induced DNA damage and subsequent cell death. Therefore, the overall therapeutic efficacy was significantly improved by cooperation with chemotherapy.

In addition to chemotherapeutic agents, immunotoxic agents, such as monoclonal antibody bevacizumab against VEGF^[^ ^179^ ^]^ and TuBB‐9 against protein Ki‐67^[^ ^180^ ^]^ (Table 2, entry 13 and 14) can be loaded into liposome to combine with PDT. A nanophotoactivatable liposome (nanoPAL) co‐loaded with PS (**BPD**) and mAb antibody (bevacizumab) was validated in a mouse model of pancreatic cancer. To enhance PDT efficacy, the antibody suppressed the VEGF‐mediated signaling pathways activated by PDT. In another study, a light‐activatable antibody conjugate, TuBB‐9‐FITC, was delivered by the liposomes to HeLa cells. After the free **BPD** and liposomal TuBB‐9‐FITC were absorbed by HeLa cells and assembled into the endosome, the endosome was disrupted by a NIR of 690 nm to release TuBB‐9‐FITC, which further targeted Ki‐67 in the nucleus followed by PDT activated by 490 nm light for cell elimination.

Immunotherapy is a popular topic for tumor therapy. NLG919, a potent indoleamine‐2,3‐dioxygenase (IDO) inhibitor, was co‐encapsulated into PpIX‐NLG@Lipo along with PS (**PpIX**) (Table 2, entry 15).^[^ ^181^ ^]^ The IDO inhibitor blocked the checkpoint by reliving the immunosuppressive microenvironment and activating the host immune system, and enhance PDT‐induced immune responses by increasing the penetration of CD8^+^ T lymphocytes into the tumor site.

Biopharmaceutical drugs are important components of chemotherapeutic drugs. Protein toxin (saprin) together with PS was delivered into MC28 fibrosarcoma cells by cell‐penetrating peptide (CPP)‐immobilized liposomes (Table 2, entry 16).^[^ ^182^ ^]^ After the liposomes were internalized by the endosome, PS was triggered to interrupt the carriers and release the cargo. Another liposomal system (Lipo (Pep, Ce6)), containing an amphiphilic and cationic D‐(KLAKLAK)_2_ peptide and **Ce6**, showed synergistic anti‐cancer activity (Table 2, entry 17).^[^ ^183^ ^]^ The membrane‐lytic D‐(KLAKLAK)_2_ peptide disrupted the endo‐lysosomal compartment and induced mitochondria‐dependent apoptosis.

The co‐encapsulation system could also be applied to the anti‐parasitic drugs miltefosine and *N*‐methyl glucamine antimoniate (Sb^V^), respectively, to cooperate with **AlClPC** to enhance the PDT in the treatment of cutaneous leishmaniasis caused by Leishmania (L.) amazonensis in C57BL/6 mice (Table 2, entry 18).^[^ ^184^ ^]^

#### 3.4.2 PDT with PTT or Magnetic Hyperthermia (MHT)

**Figure 18:**
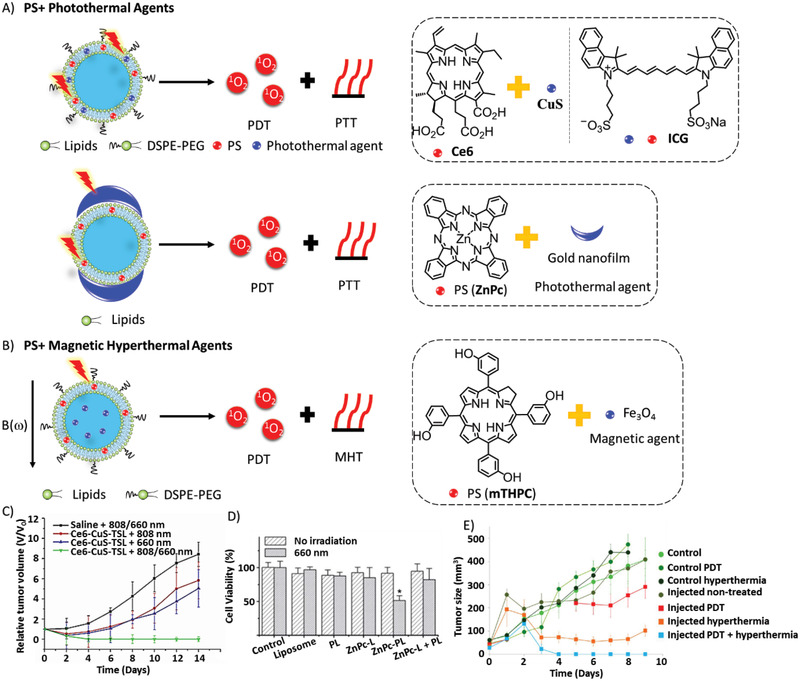
Combination of PDT with A) PTT excited by light or B) MHT excited by magnetic field. The therapeutic effects of C) Ce6‐CuS‐TSL liposomes. Reproduced with permission.^[^ ^188^ ^]^ Copyright 2016, Elsevier. D) Plasmonic liposomes (PLs). Reproduced with permission.^[^ ^189^ ^]^ Copyright 2014, Royal Society of Chemistry. E) Ultramagnetic photosensitive liposomes (UMPL). Reproduced with permission.^[^ ^191^ ^]^ Copyright 2015, American Chemical Society.

As a non‐invasive and non‐resistant therapy, PTT has been applied to treat various types of tumors by converting light energy to heat, thereby increasing the target cell temperature beyond the threshold to trigger cell death.^[^ ^185^ ^]^ In clinic and clinical trials, the combination of PTT and PDT has demonstrated the synergistic effects in tumor therapy.^[^ ^186^ ^]^ Liposome simultaneously encapsulating PSs and photothermal agents are excellent nano‐platform for combination of PDT and PTT (**Figure** **18**A). Photothermal effects also assist in the disruption of photothermal‐responsive liposomes for drug release.

Most photothermal agents are inorganic materials as they exhibit high stability, high absorption efficiency, high photothermal effect, and activation by NIR light irradiation.^[^ ^187^ ^]^ For example, folate‐conjugated thermo‐sensitive liposomes (Ce6‐CuS‐TSL) co‐loaded with **Ce6** and copper sulfide (CuS) as the photothermal agent, were prepared by Li et al. (Figure 18A–C).^[^ ^188^ ^]^ The PS was released by the photothermal effect‐induced disruption of liposomes. In plasmonic liposomes (ZnPc‐PLs), gold nanofilm for PTT was coated on the surface of liposomes encapsulating **ZnPc** for PDT to enhance therapeutic effects on cancer cells.^[^ ^189^ ^]^ (Figure 18A–D) However, to solve the leakage of toxic heavy metal, liposomes facilitated the integration of CuInS_2_/ZnS nanocrystals, which exhibited PDT and PTT effects, with rGO to build CuInS_2_/ZnS/liposome‐rGO nanocomposites.^[^ ^99^ ^]^ rGO nanosheets are responsive to PTT and absorbs the leaking heavy metal Cu^2+^ ions. This strategy achieved tumor suppression and low toxicity.

As the only FDA‐approved NIR dye, **ICG** can be used for combined PDT/PTT. However, it has poor photo‐stability, poor stability in blood, and especially lacks tumor targeting. To overcome these problems, multi‐functional liposomal formulations of **ICG** with other recognition moieties, such as iRGD‐ICG‐LPs,^[^ ^161^ ^]^ ICG‐MCL,^[^ ^120^ ^]^ and HA‐PEG‐MPLs^[^ ^190^ ^]^ have been developed (Figure 18A). Liposome iRGD‐ICG‐LPs modified with iRGD were specifically internalized into targeted cancer cell via the interactions between iRGD peptide and *α*_v_ *β*_3_ integrin. The photothermal image showed that the temperature in the tumor regions rapidly increased to 51.7 °C and remained stable upon laser irradiation. Cell‐like liposomes (ICG‐MCLs) were reconstructed from the cell membrane of native glioma cells, which were specifically internalized by homogenous tumor cells. To deliver HA‐PEG‐MPLs to tumor cells site‐specifically, iron oxide magnetic nanoparticles in the aqueous core are responsive to magnetic targeting in a magnetic field, and HA‐PEG on the liposome is responsive to ligand targeting by CD44 receptors on tumors. In general, dual functional liposomal **ICG**s for combined PTT/PDT can efficiently suppress tumor progression.

Unlike the photothermal effect, MHT also exhibits anti‐tumor effects. Wilhelm et al. reported ultramagnetic photosensitive liposomes (UMPL) loaded with **mTHPC** and iron oxide nanoparticles for MHT (Figure 18B–E).^[^ ^191^ ^]^ The combined treatments achieved higher efficiency in tumor depletion than each monotherapy.

#### 3.4.3 Combination of Different PDTs

**Figure 19:**
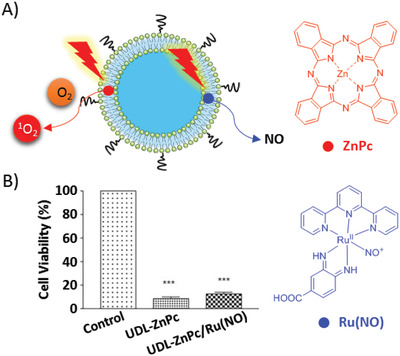
A) Structure of liposome co‐encapsulated with **ZnPC** and **Ru(NO)** and the activation modes via two photosensitive mechanisms. B) In vitro phototoxicity against B16‐F10 cells. Reproduced with permission^[^ ^192^ ^]^ Copyright 2017, Elsevier.

Although the effects of oxygen‐dependent type II PDT are severely limited by hypoxia, excess reactive species nitric oxide (NO) can induce tumor damage under hypoxic conditions. For example, the nitrosyl ruthenium complex [Ru(NH.NHq)(tpy)NO]^3+^ (**RuNO**) as a NO donor was co‐encapsulated with **ZnPc** into ultradeformable liposomes (UDLs), and showed higher flexibility and greater skin‐penetrating ability than conventional liposomes (**Figure** **19**).^[^ ^192^ ^]^ This strategy of combined PDT efficiently destroyed skin melanoma cells by NIR irradiation (675 nm).

**Figure 20:**
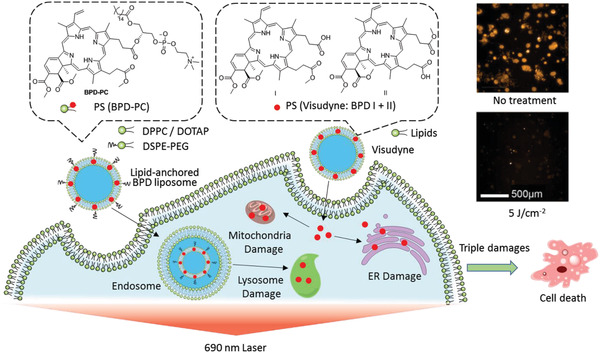
Schematic illustration of targeted PDT to three organelles (lysosome, mitochondria and ER) by lipid‐anchored **BPD** liposome and Visudyne. Reproduced with permission.^[^ ^61^ ^]^ Copyright 2019, Wiley‐VCH.

The efficiency of PDT may differ due to the differences in cellular localization and mechanism of action of different PSs, leading to subcellular‐component‐selective photodamage. For example, Visudyne (clinically approved liposomal **BPD**) targets mitochondria and ER, whereas the liposomal **PC‐BDP** targets the lysosome (**Figure** **20**).^[^ ^61^, ^193^ ^]^ Therefore, this strategy involved that the simultaneous targeted PDTs to three organelles by a single wavelength of light considerably enhanced PDT.

#### 3.4.4 PDT with Chemodynamic Therapy (CDT)

**Figure 21:**
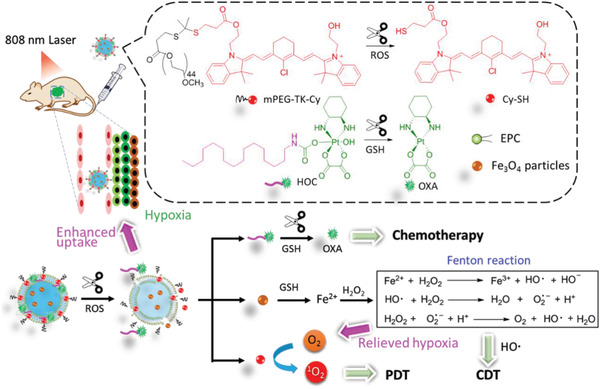
Schematic illustration of the synthesis of RALP@HOC@Fe_3_O_4_ liposome and i.v. injection. The collapse of ROS‐sensitive liposome induced the release of photo/chemodynamic therapeutic agents which were then activated by TME.

In contrast to oxygen‐dependent PDT, CDT converts endogenous H_2_O_2_ to ROS via the Fenton reaction. A theranostic system RALP@HOC@Fe_3_O_4_ based on ROS‐activatable liposomes (RALP) regulated TME to enhance synergistic PDT and CDT (**Figure** **21**).^[^ ^105^ ^]^ Upon PS **Cy** was irradiated by NIR (808 nm), the laser source activated the cleavage reaction between ROS‐liable thioketal bond of mPEG‐TK‐Cy and PEG corona, thereby enhancing tumor cell uptake. In addition, the ROS‐sensitive lipid EPC with a double bond was oxidized to release liposomal cargoes, such as Fe_3_O_4_ and the prodrug hexadecyl‐oxaliplatin carboxylic acid (HOC). Low‐toxicity HOC and Fe_3_O_4_ were reduced to highly toxic OXA and Fe^2+^ by endogenous GSH, respectively. The consumption of GSH promotes the generation of H_2_O_2_, which is converted to ROS product (•OH) and oxygen. The ROS product (•OH) potentially induced liposome collapse and cell damage. Semi‐quantitative analysis of tumor oxygen saturation level (sO_2_ average total) by PA imaging revealed that tumor hypoxia was relieved by RALP for PDT. NIR fluorescence imaging revealed the efficient accumulation of RPLR at the tumor sites by the EPR effect.

### 3.5 Synergistic Combination of PDT with Multi‐Modes of Theranostics

Liposomes can serve as a nano‐platform for multi‐modes of theranostics. Single imaging methods have limitations in terms of resolution, depth penetration, signal‐to‐noise ratio, and anatomy information, whereas their synergism can address these limitations.

**Figure 22:**
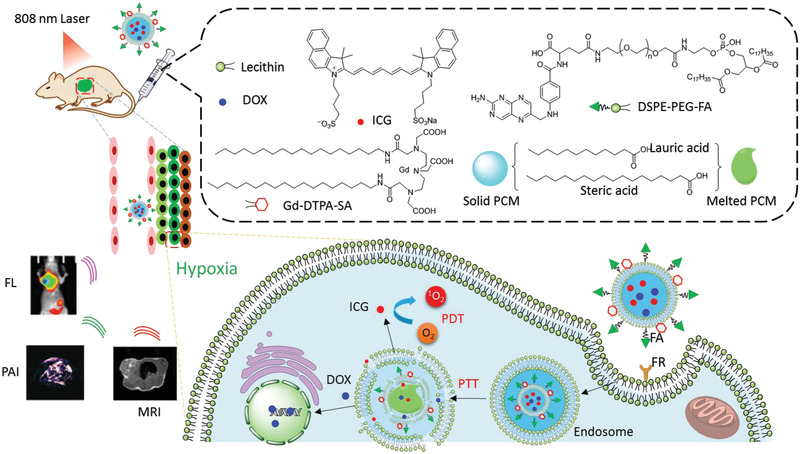
Schematic illustration of the multi‐diagnoses (MRI, FLI and PAI) and multi‐therapies (PDT, chemotherapy and PTT) based on multi‐functional theranostic liposomes. Reproduced with permission.^[^ ^194^ ^]^ Copyright 2019, American Chemical Society.

Fan et al. developed multi‐functional theranostic nano‐platforms based on thermo‐sensitive liposomes triggered by NIR irradiation (**Figure** **22**).^[^ ^194^ ^]^ NIR irradiation induced thermo‐sensitive liposomes and phase‐change materials (PCM) to release drugs. The nano‐platform is capable of treating tumor by combined therapies (chemotherapy, PTT and PDT) and triple‐modal imaging (FLI, PA and MRI)‐guided therapies. Later, a similar platform loaded TPZ as a hypoxia‐activated prodrug for combined therapies.^[^ ^195^ ^]^

**Figure 23:**
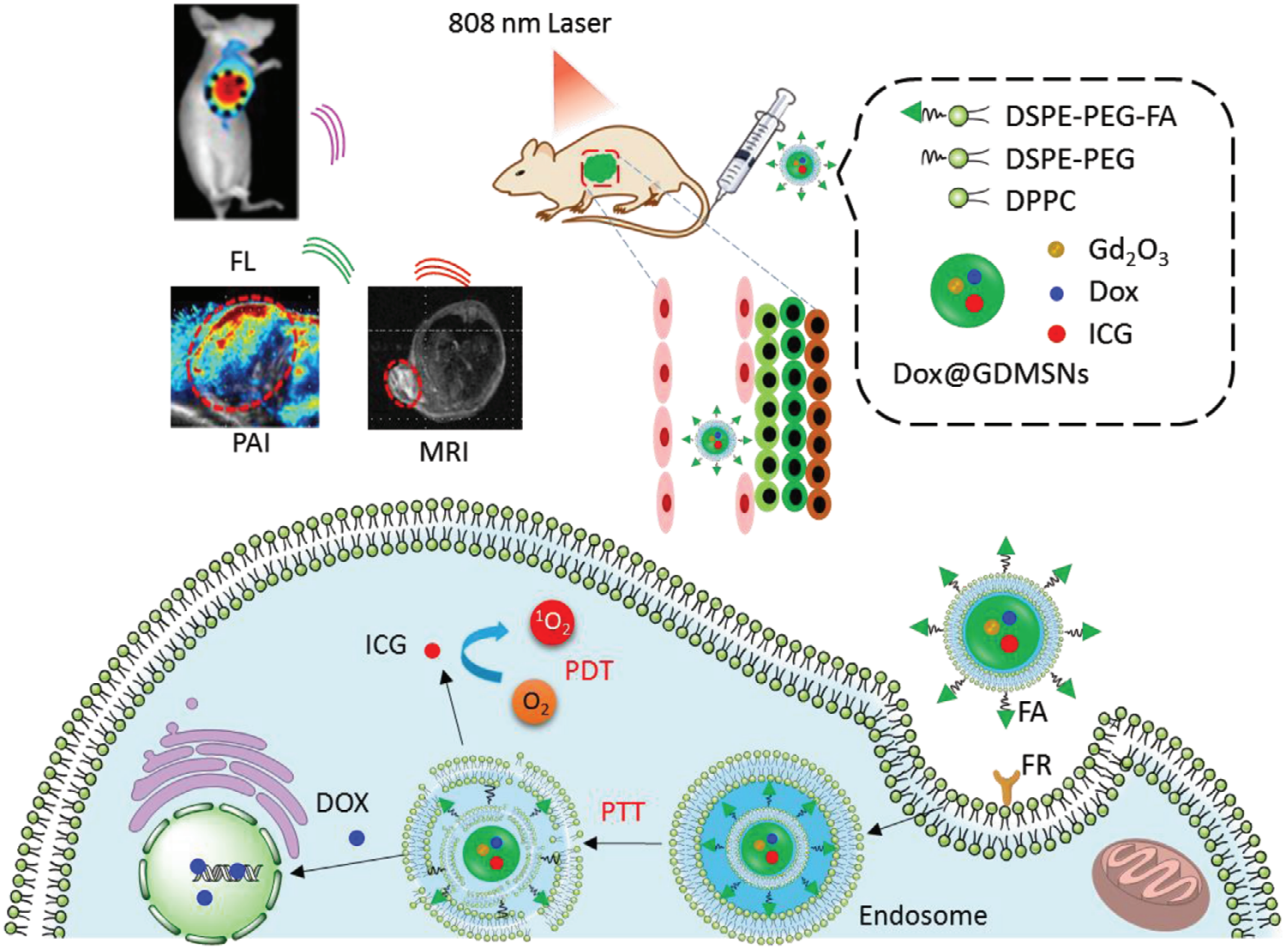
Schematic illustration of the preparation of multi‐functional theranostic nanocomposite DOX@GdMSNs‐ICG‐TSLs for fluorescence/photoacoustic/magnetic resonance imaging‐guided chemo‐ and phototherapies. Reproduced with permission.^[^ ^30^ ^]^ Copyright 2018, American Chemical Society.

Li et al. constructed a theranostic hybrid nano‐platform consisting of liposome‐conjugated mesoporous silica nanoparticles for triple‐model imaging‐guided synergistic cancer therapy (**Figure** **23**).^[^ ^30^ ^]^ To overcome the limitations in dispersibility and drug leakage of mesoporous silica nanoparticles (MSNs) containing **ICG**, contrast agent Gd, and chemotherapeutic agent Dox, a thermo‐sensitive liposome was fabricated on the surface of MSNs. **ICG** in liposomes was responsive to release of cargoes by photothermal effect upon NIR irradiation. Folic acid‐modified liposomes improved tumor‐specific cellular uptake. Using this theranostic platform, NIRF/PA/MRI trimodal imaging successfully visualized the distribution of liposomes and guided chemotherapy and phototherapy for the depletion of tumors.

**Figure 24:**
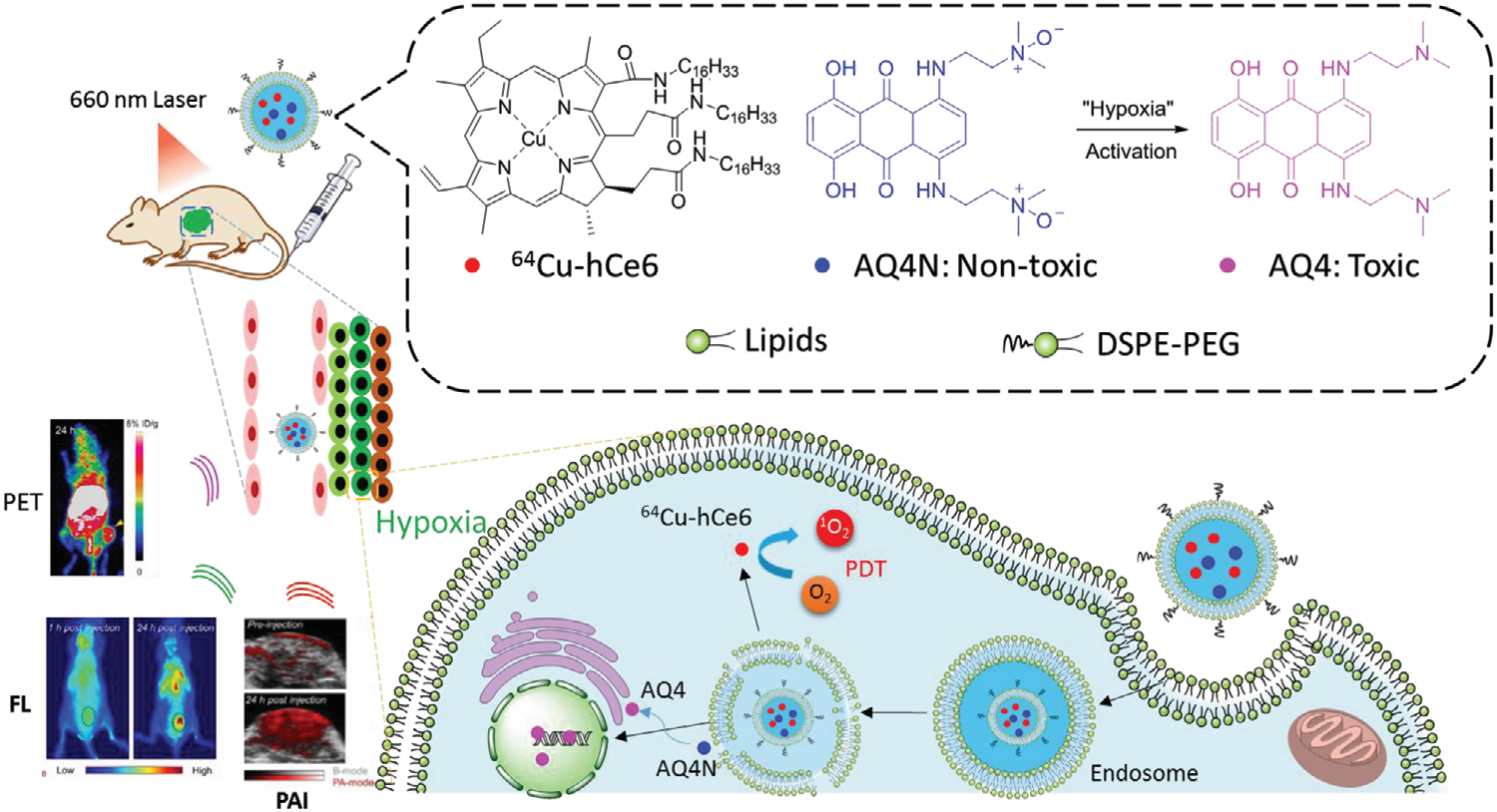
Schematic illustration of components of AQ4N‐hCe6‐liposome and its applications in tumor theranostic. Reproduced with permission.^[^ ^160^ ^]^ Copyright 2017, American Chemical Society.

Liu reported a liposome‐based theranostic nanomedicine that could improve therapeutic efficacy by activating chemotherapy triggered by PDT‐induced hypoxia (**Figure** **24**).^[^ ^160^ ^]^ At the same time, **Ce6** chelated with the PET tracer ^64^Cu was capable of guiding tumor therapy by PET, PA, and FL imaging. The liposome platform showed several advantages, including synergistic treatment against hypoxia, multi‐model imaging for real‐time in vivo monitoring, and excellent biocompatibility.

**Figure 25:**
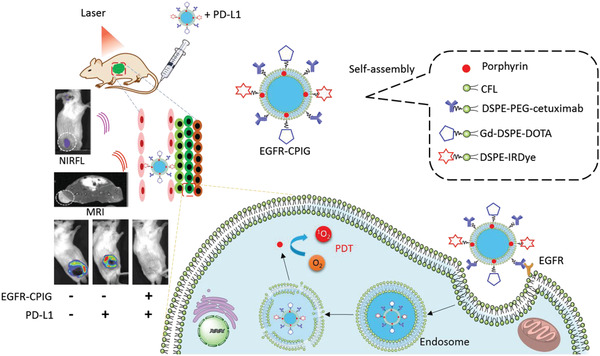
Schematic illustration of components and preparation of EGFR‐CPIG and its application of theranostic in vivo. Reproduced with permission.^[^ ^156^ ^]^ Copyright 2018, Royal Society of Chemistry.

EGFR‐targeted liposomal nanohybrid cerasomes performed excellent theranostic functions and immune checkpoint inhibition in a mouse model of colorectal cancer (**Figure** **25**).^[^ ^156^ ^]^ The cerasome PD‐L1‐PCI‐Gd contains PS (**porphyrin**), T1 MRI contrast (Gd‐DSPE‐DOTA), NIR dye (DSPE‐IRDye800CW), and EGFR antibody (DSPE‐PEG‐cetuximab). The platform integrated the good biocompatibility of liposomes and the high stability of silica. The combined therapies of PD‐L1 immunotherapy and PDT showed synergistic therapeutic effects to achieve superior therapeutic efficacy and prevent tumor recurrence.

## 4 Conclusion and Perspectives

Besides optimizing intrinsic characteristics of PSs toward high efficacy, safety, better penetration, and greater effectivity against TME as the fundamental approaches to improve PDT, improvements in drug delivery and combined therapy are alternative options. In this review, liposomes have exhibited a promising nano‐platform for enhancing PDT. Compared with conventional liposomes, functionalized liposomes offer more advantages in therapeutic outcomes. The flexibility in surface modification and payloads improves PDT stability, therapeutic efficiency, and specificity. However, to translate their applications in the clinical practice, there are still some key points awaiting improvement.

Owing to their promising properties, liposomal formulations might be potential to help more PSs with good photosensitivity, to overcome their typical drawbacks that hinder further biomedical applications. The liposome is also suitable for free‐radical therapy like type I PDT, in the manner of oxygen‐independence.^[^ ^196^ ^]^ In another case, a liposomal system that generates cytotoxic ROS, such as artemisinin, is also potential in cancer therapy.^[^ ^197^ ^]^ From a safety point of view, apart from conventional components for liposomes, natural or biocompatible carriers have great potential in biomedical applications of PDT. A sperm‐driven micromotor delivered DOX to cancer cells.^[^ ^198^ ^]^ Liposome‐like hollow nanoparticles emerge as a similar multi‐functional theranostic platform.^[^ ^199^ ^]^

Targeted therapy and personal medicine are key concepts in drug development. To improve the specificity and safety, target‐specific delivery and controllable/triggerable release of liposomal cargos on demand are still big challenges in drug delivery. Cytochrome c/cardiolipin complex can facilitate the release of liposomal cargoes by peroxidative permeabilization.^[^ ^200^ ^]^ To date, only one example of a micromotor based‐on red blood cell has been reported to enhance PDT.^[^ ^25^ ^]^ It has the potential to develop liposome‐based robots to deliver PS and other anti‐cancer drugs to tumor sites, since nano‐ and micromotors have experienced rapid development and shown promising potential in related biomedical applications.^[^ ^201^ ^]^

Due to the limited tissue‐penetration ability of visible light and NIR in the human body, an alternative strategy for the remote‐activation of liposomal PS is urgently needed. For instance, X‐ray‐induced PDT combined with radiotherapy has been investigated.^[^ ^202^ ^]^ Sonodynamic therapy^[^ ^203^ ^]^ has a similar damage mechanism to PDT and the potency in liposome‐based drug delivery.^[^ ^204^ ^]^ Compared with PDT, sonodynamic therapy has unlimited tissue penetration ability for sono‐triggered drug release and sono‐activation of sensitizers.

The identification of more imaging tools and therapeutic agents that can combine with PDT for image‐guided liposomal theranostic and combined therapies is also needed. Radiolabeled liposomes can be used in theranostics, such as radiotherapy and diagnosis (SPECT/PET).^[^ ^205^ ^]^ Raman scattering spectroscopy has been used for PDT via AuNS‐DTDC@SiO_2_‐PpIX‐TAT.^[^ ^206^ ^]^ Commercial available Lipofectamine has shown excellent delivery efficiency of RNA and plasmid DNA by lipofection. The liposome‐based combined therapies of PDT and gene therapy could be expected reasonably, since ROS‐responsive liposomes as biocompatible nano‐carrier, have successfully delivered gene silencing reagents (antisense oligonucleotide) for gene knockdown,^[^ ^126^, ^207^ ^]^ gene editing system (CRISPR‐Cas9) for gene knockout^[^ ^208^ ^]^ and plasmid DNA (pDNA) for transfection.^[^ ^209^ ^]^

Taken together, liposomes, as a multi‐functional theranostic platform for PDT, have achieved rapid progress in recent years. We believe this review will be valuable for researchers studying the drug delivery of liposome‐based PDT and that there will be further discoveries in this area in the future.

## Conflict of Interest

The authors declare no conflict of interest.
